# Selective Formation, Reactivity, Redox and Magnetic Properties of Mn^III^ and Fe^III^ Dinuclear Complexes with Shortened Salen-Type Schiff Base Ligands

**DOI:** 10.3390/ijms21217882

**Published:** 2020-10-23

**Authors:** Luca Rigamonti, Paolo Zardi, Stefano Carlino, Francesco Demartin, Carlo Castellano, Laura Pigani, Alessandro Ponti, Anna Maria Ferretti, Alessandro Pasini

**Affiliations:** 1 Dipartimento di Scienze Chimiche e Geologiche, Università degli Studi di Modena e Reggio Emilia, via G. Campi 103, 41125 Modena, Italy; laura.pigani@unimore.it; 2 Dipartimento di Chimica, Università degli Studi di Milano, via C. Golgi 19, 20133 Milano, Italy; paolo.zardi@unipd.it (P.Z.); stefano04.85@gmail.com (S.C.); francesco.demartin@unimi.it (F.D.); carlo.castellano@unimi.it (C.C.); alessandro.pasini@unimi.it (A.P.); 3 Dipartimento di Scienze Chimiche, Università degli Studi di Padova, via Francesco Marzolo 1, 35131 Padova, Italy; 4 Istituto di Scienze e Tecnologie Chimiche “Giulio Natta”, Consiglio Nazionale delle Ricerche (SCITEC-CNR), via G. Fantoli 16/15, 20138 Milano, Italy; alessandro.ponti@scitec.cnr.it (A.P.); anna.ferretti@scitec.cnr.it (A.M.F.)

**Keywords:** Schiff base ligands, manganese(III), iron(III), dinuclear complexes, X-ray structures, magnetic properties, exchange interaction, hydrolysis, cyclic voltammetry

## Abstract

The reactivity of the shortened salen-type ligands H_3_salmp, H_2_salmen and H_2_sal(*p*-X)ben with variable *para*-substituent on the central aromatic ring (X = *t*Bu, Me, H, F, Cl, CF_3_, NO_2_) towards the trivalent metal ions manganese(III) and iron(III) is presented. The selective formation of the dinuclear complexes [M_2_(μ-salmp)_2_], M = Mn (**1a**), Fe (**2a**), [M_2_(μ-salmen)_2_(μ-OR)_2_)], R = Et, Me, H and M = Mn (**3a**–**c**) or Fe (**4a**–**c**), and (M_2_(μ-sal[*p*-X]ben)_2_(μ-OMe)_2_), X = *t*Bu, Me, H, F, Cl, CF_3_, NO_2_ and M = Mn (**5a**–**g**) or Fe (**6a**–**g**), could be identified by reaction of the Schiff bases with metal salts and the base NEt_3_, and their characterization through elemental analysis, infrared spectroscopy, mass spectrometry and single-crystal X-ray diffraction of **2a**·2AcOEt, **2a**·2CH_3_CN and **3c**·2DMF was performed. In the case of iron(III) and H_3_salmp, when using NaOH as a base instead of NEt_3_, the dinuclear complexes [Fe_2_(μ-salmp)(μ-OR)(salim)_2_], R = Me, H (**2b**,**c**) could be isolated and spectroscopically characterized, including the crystal structure of **2b**·1.5H_2_O, which showed that rupture of one salmp^3−^ to two coordinated salim^−^ ligands and release of one salH molecule occurred. The same hydrolytic tendency could be identified with sal(*p*-X)ben ligands in the case of iron(III) also by using NEt_3_ or upon standing in solution, while manganese(III) did not promote such a C–N bond breakage. Cyclic voltammetry studies were performed for **3b**, **4b**, **5a** and **6a**, revealing that the iron(III) complexes can be irreversibly reduced to the mixed-valence Fe^II^Fe^III^ and Fe^II^_2_ dinuclear species, while the manganese(III) derivatives can be reversibly oxidized to either the mixed-valence Mn^III^Mn^IV^ or to the Mn^IV^_2_ dinuclear species. The super-exchange interaction between the metal centers, mediated by the bridging ligands, resulted in being antiferromagnetic (AFM) for the selected dinuclear compounds **3b**, **4b**, **5a**, **5e,** **5f**, **6a** and **6e**. The coupling constants *J* (–2*J* **Ŝ**_1_**·Ŝ**_2_ formalism) had values around −13 cm^−1^ for manganese(III) compounds, among the largest AFM coupling constants reported so far for dinuclear Mn^III^_2_ derivatives, while values between −3 and −10 cm^−1^ were obtained for iron(III) compounds.

## 1. Introduction

**Figure 1:**
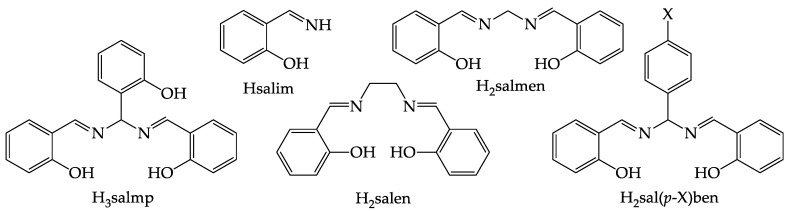
Pentadentate N_2_O_3_ (H_3_salmp), tetradentate N_2_O_2_ (H_2_salen, H_2_salmen and H_2_sal(*p*-X)ben, X = *t*Bu, Me, H, F, Cl, CF_3_, NO_2_) and bidentate NO (Hsalim) Schiff bases.

Salen-related [1] Schiff bases bearing only one carbon atom between the two iminic C=N bonds of the condensed salicylaldehyde (salH) moieties are known as H_3_salmp (*N,N*′-*bis*(salicylidene)-2-hydroxyphenylmethanediamine), H_2_salmen (*N,N*′-*bis*(salicylidene)methanediamine) and H_2_salben (*N,N*′-*bis*(salicylidene)phenylmethanediamine) (Figure 1). H_3_salmp can be easily obtained by the reaction of three moles of salH and two moles of ammonia [2], and there are reports of its synthesis since the beginning of the 20th century [3]. H_2_salben’s derive from H_3_salmp via substitution of the central salH moiety by reaction with variably substituted benzaldehydes [4], while H_2_salmen can be obtained by reaction of salH with either methanediamine [5], formaldehyde and ammonia [6] or hexamethylenetetramine [7]. Even if some reports indicate mononuclear complexes with a metal:ligand (M:L) 1:1 ratio [8,9,10,11,12,13,14,15,16,17] as H_2_salen [18], such shortened ligands preferentially form oligonuclear metal complexes [19,20] due to the tension of the four-membered MNCN metallacycle in a hypothetic mononuclear compound. To the best of our knowledge, there is only one reported X-ray structure in which the two iminic nitrogen atoms coordinate to the same metal ion, (Zr(Hsalmp)_2_) [21]. However, in this compound the M:L ratio is 1:2 and the zirconium(IV) ion is octacoordinated with rather long bond distances (2.1–2.4 Å), which lowers the tension given by the four-membered metallacycle.

With the exception of H_3_salmp, for which quite a few complexes have been synthesized and structurally characterized up to date [10,21,22,23,24,25,26,27,28,29,30], less is known about the other two classes of shortened Schiff base derivatives [7,11,19,20,31]. Nevertheless, it is undeniable the usefulness of all these ligands for the synthesis of efficient catalysts [15,16,17], their use for metal sequestration in solution [32], synthesis of biomimetic systems [24] and the fascinating magnetic properties of the corresponding oligonuclear clusters [19,20,23,24,26,27,28,31,33]. Within this last topic, at the beginning of the new Millennium we started a systematic study on the coordination ability of H_2_salmen and H_2_salben with divalent metal ions [11] like copper(II) [19] and cobalt(II) [20,31], and the possibility to oxidize the latter to cobalt(III) and additionally form mixed-valence oligonuclear clusters [20,31]. In this work we aim to describe in detail the reactivity of these ligands toward manganese(III) and iron(III) in order to enrich the scenario with the coordination ability of H_3_salmp [26,27,28,34], H_2_salmen and H_2_salben towards these two trivalent ions. In particular, the formation of dinuclear complexes is confirmed by single-crystal X-ray diffraction structural studies on several derivatives. Furthermore, cyclic voltammetry (CV) measurements on selected so-obtained dinuclear complexes (Table 1) are presented in order to understand the redox properties of the metal ions conferred by the ligands, together with the temperature dependence of their magnetic moment, providing quantitative insight into the super exchange interaction between the bridged metal ions.

## 2. Results and Discussion

### 2.1. Synthesis of the Shortened Schiff Bases and Crystal Structures of H_2_sal(p-X)ben (X = tBu, CF_3_)

**Figure 2:**
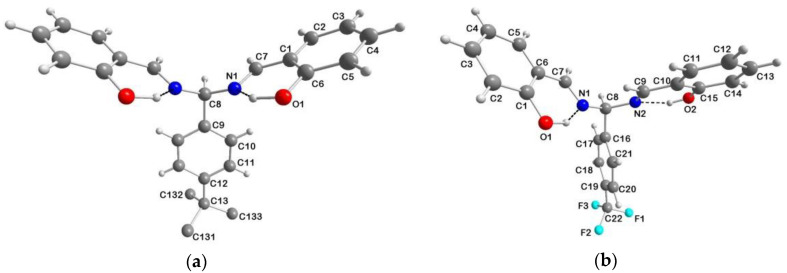
Crystal structures of (**a**) H_2_sal(*p*-*t*Bu)ben and (**b**) H_2_sal(*p*-CF_3_)ben with atom numbering; color code: O = red, N = blue, C = grey, H = white, F = turquoise, intramolecular H-bond = dashed black line. Molecule (**a**) is located on a crystallographic mirror plane passing through atoms C8, C9, C12 and C13, and the *t*Bu group is disordered (only one conformation is shown).

The Schiff bases H_3_salmp [2], H_2_salmen [5] and H_2_sal(*p*-X)ben (X = *t*Bu, Me, H, F, Cl, CF_3_, NO_2_) [4,11,19,20,31] (Figure 1), used in this paper as ligands for manganese(III) and iron(III) (Table 1), were obtained following the synthetic procedures reported in the literature. Some of them have been structurally characterized and previously described in the literature; in particular, the X-ray crystal structures of H_2_sal(*p*-NO_2_)ben [31], H_2_salmen [6] and H_3_salmp [35] are available. We could obtain good crystals suitable for X-ray diffraction of the compounds H_2_sal(*p*-*t*Bu)ben and H_2_sal(*p*-CF_3_)ben by slow evaporation of their chloroform solutions. The crystal structures could be solved, and the perspective views of these molecules are shown in Figure 2. Bond lengths and angles are compatible with Schiff base structures derived from salH and diamines where the enolimine tautomer is favored over the ketoamine form [6,31,35].

The H_2_sal(*p*-*t*Bu)ben molecule is located on a crystallographic mirror plane, passing through atoms C8, C9, C12 and C13. This leads the *t*Bu group to be disordered with two different conformations having occupancy of 0.5 each. No relevant intermolecular interactions below the sum of the van der Waals radii were observed in the crystal packing. The H_2_sal(*p*-CF_3_)ben molecule instead showed significant C–H···F intermolecular interactions as depicted in Figure S1 in Supplementary Materials, where a portion of the crystal packing is reported. In both cases, the molecules adopted a conformation mainly determined by the presence of strong intramolecular O–H···N hydrogen bonds (H-bonds) involving the phenolic groups and the iminic nitrogen atoms of the same sal moiety of the molecule (O1–HO1···N1, *r*_O__···N_ = 2.608(2) Å, OH···N angle = 138° in H_2_sal(*p*-*t*Bu)ben, and O1–HO1···N1 and O2–HO2···N2, *r*_O__···N_ = 2.612(9) and 2.608(9) Å, respectively, with an average OH···N angle = 147° in H_2_sal(*p*-CF_3_)ben).

### 2.2. Synthesis, Isolation and Reactivity of Dinuclear Complexes with H_3_salmp

As stated above, the most studied ligand for complexation of iron(III) and manganese(III) is H_3_salmp, where M^III^_2_ complexes have been isolated, M = Mn (**1a**) and Fe (**1b**), together with their mixed-valence M^II^M^III^ and reduced M^II^_2_ dinuclear species [26,27,28,34]. Oxidation to Mn^III^Mn^IV^ and Mn^IV^_2_ has also been achieved through cyclic voltammetry [27]. Due to the rich chemistry and the range of accessible oxidation states, we then decided to start our synthetic and reactivity studies by obtaining **1a** and **2a** while modifying the reaction conditions.

Instead of using MnCl_2_, salH and NH_3_ in boiling EtOH with ligand assembly directly in the reaction mixture followed by metal oxidation in air [27], H_3_salmp was first isolated, suspended in MeOH and NEt_3_ as a deprotonating agent and reacted with Mn(AcO)_3_·2H_2_O, Equation (1). After a few hours at room temperature (RT), **1a** was isolated as a dark brown powder with two cocrystallized water molecules, whose elemental analysis, infrared and mass spectra confirmed the intactness of the ligand and the dinuclear nature of the complex. Substitution of MeOH with DMF as solvent caused a lowering in the yield of isolated product, together with cocrystallized DMF in substitution of water. This was confirmed by both elemental analysis and infrared spectroscopy, where the C=O stretching band of DMF at 1663 cm^−1^ was present [36,37,38]. Furthermore, the synthesis was also tested without the addition of NEt_3_, thanks to the role of the acetate anion as base, and the dinuclear species **1a** was also suitably isolated, even if in lower yields.

2 H_3_salmp + 2 MX_3_ + 6 Et_3_N → [M_2_(μ-salmp)_2_] + 6 Et_3_NHX M = Mn (**1a**), Fe (**2a**); X = AcO, Cl, NO_3_ (1)

The same results were obtained in the case of iron(III) (either chloride or nitrate salt) with the isolation of **2a** as red-brown solid, Equation (1), similar to what has been reported in the literature using boiling EtOH [34]. **2a** also cocrystallized with H_2_O or DMF molecules, and especially in the latter case this was clearly detected from the infrared spectrum with the C=O stretching of DMF at 1668 cm^−1^. Use of CH_3_CN as solvent was also tested in this case, causing the undesired coprecipitation of Et_3_NHCl salt together with the product, which forced extensive washing of the obtained solid with a MeOH:H_2_O 1:1 mixture. Nevertheless, all isolated compounds showed *m/z* signals at 799, 821 and 1618, attributable to [M + 1]^+^, [M + Na]^+^ and [2M + Na]^+^ ions, respectively, diagnostic of the intact dinuclear species [Fe_2_(μ-salmp)_2_]. Crystallization of the reaction mixtures in DMF and CH_3_CN with AcOEt and *i*Pr_2_O, respectively, yielded crystals of **2a**·2AcOEt and **2a**·2CH_3_CN suitable for single-crystal X-ray diffraction studies (see below), further confirming the invariability of the obtained dinuclear species.

The use of NaOH in substitution of NEt_3_ as a base in the reaction did not alter the isolation of the dinuclear manganese(III) **1a**, while it invariably caused the rupture of one salmp^3−^ to two salim^−^ (Figure 1) fragments with iron(III), and substitution of the central phenoxido bridging oxygen atom with a methoxido or hydroxido bridging ligand, depending whether the reaction was conducted in MeOH or DMF, respectively, obtaining **2b** or **2c**, Equation (2). The reaction was not tested in CH_3_CN due to the concomitant precipitation of Et_3_NHCl. The coordinated salim^−^ ligands were immediately recognized by infrared spectroscopy, with the presence of N–H stretching in the spectrum as a narrow band at 3302 cm^−1^. The C=N stretching also shifted to 1615 cm^−1^, probably due to the change in the coordination environment (see below) and the presence of two different C=N bonds (salmp^3−^ and salim^−^). Mass spectra of both **2b** and **2c** showed the main peaks at *m/z* 749 and 606 ascribable to [Fe_2_(salmp)(salim)_2_(OMe) + Na]^+^ and [Fe_2_(salmp)(salim)(OMe)]^+^ ions, which undoubtedly proved the new dinuclear species obtained. The absence of any difference between the two compounds was safely ascribed by the fact that the mass spectra were registered in MeOH, which caused a rapid OH ↔ OMe scrambling in solution. We also obtained single crystals of **2b**·1.5H_2_O suitable for X-ray diffraction by diffusion of *i*Pr_2_O into the methanolic reaction mixture, confirming the structure of the dinuclear compound (see below).

2 H_3_salmp + 2 FeCl_3_ + 6 NaOH + MeOH → [Fe_2_(μ-salmp)(μ-OMe)(salim)_2_] (**2b**) + salH + 6 NaCl + 5 H_2_O (2)

### 2.3. Dinuclear Complexes with Tetradentate Ligands and Reactivity in Solution

Moving to the tetradentate ligands, reaction of manganese(III) acetate and iron(III) chloride with H_2_salmen was performed in three different solvents, EtOH, MeOH or DMF. In the absence of the central phenoxido unit provided by salmp^3−^, the dinuclear species [M_2_(μ-salmen)_2_(μ-OR)_2_], M = Mn, **3a**–**c**, and Fe, **4a**–**c**, with R = Et, Me and H, respectively, were obtained, Equation (3). The fifth coordinating oxygen atom of the pentadentate salmp^3−^ was here replaced by the bridging alkoxido/hydroxido groups, while maintaining the same dinuclear structure. The bridging OH^−^ groups in **3c** and **4c** should be provided either by the crystallization water molecules of Mn(AcO)_3_·2H_2_O or by the amount of water present in the solvent when operating in DMF, especially in the case of the iron(III) derivative. This was also confirmed by the crystallization of **3b** from DMF-*i*Pr_2_O, which yielded crystals of **3c**·2DMF, where the OMe ↔ OH scrambling occurred within the crystallization time (see below). Together with the elemental analyses, the composition of these complexes was confirmed by mass spectrometry; for example, peaks at *m/z* 727 and 729, even if weak, due to [Mn_2_(salmen)_2_(OEt)_2_ + Na]^+^ and [Fe_2_(salmen)_2_(OEt)_2_ + Na]^+^ ions, were observed in the case of **3a** and **4a**, respectively, together with signals of species obtained by the OEt ↔ OMe scrambling in methanolic solutions, such as [M_2_(salmen)_2_(OMe)]^+^ at *m/z* 645 and 647 for M = Fe and Mn, respectively.

2 H_2_salmen + 2 MX_3_ + 2 ROH + 6 Et_3_N → [M_2_(μ-salmen)_2_(μ-OR)_2_] + 6 Et_3_NHX R = H, Me, Et; M = Mn (**3a**–**c**), Fe (**4a**–**c**); X = AcO, Cl (3)

Reactivity of H_2_sal(*p*-X)ben with manganese(III) acetate in MeOH and Et_3_N revealed to be superimposable with the case of H_2_salmen, and the series [Mn_2_(sal(*p*-X)ben)_2_(OMe)_2_], **5a**–**g**, with X = *t*Bu, Me, H, F, Cl, CF_3_, NO_2_, respectively, was obtained in acceptable yields, Equation (4). Infrared spectra showed the C=N stretching around 1620 cm^−1^, with no modulating effect by the *para* substituent X, as expected for the absence of conjugation with the C=N bonds. Mass spectra displayed the most intense peaks at *m/z* corresponding to [M – OMe]^+^ species, together with [M + 1]^+^ and [M + Na]^+^ signals at variable intensities, which guaranteed the integrity of the dinuclear complexes.

2 H_2_sal(*p*-X)ben + 2 MX_3_ + 2 MeOH + 6 Et_3_N → [M_2_(μ-sal(*p*-X)ben)_2_(μ-OMe)_2_] + 6 Et_3_NHX X = *t*Bu, Me, H, F, Cl, CF_3_, NO_2_; M = Mn (**5a**–**g**), Fe (**6a**–**g**); X = AcO, NO_3_ (4)

In the case of the reaction of iron(III) salts (mostly nitrate due to the easier handling compared to FeCl_3_) with H_2_sal(*p*-X)ben, as already shown by reaction with H_3_salmp in the presence of NaOH, the tendency of the dinuclear complexes [Fe_2_(sal(*p*-X)ben)_2_(OMe)_2_], **6a**–**g**, to hydrolyze directly in the reaction mixture, even working with NEt_3_, was enhanced. In fact, we isolated solid phases with good purity of only **6a** (*t*Bu), **6c** (H) and **6e** (Cl) by adjusting the reaction time to about 1 h, Equation (4). The mass spectra of freshly prepared methanolic solutions of the three compounds showed mass peaks attributable to [M – OMe]^+^, [M + 1]^+^ and [M + Na]^+^ positive ions, as sign of the structural integrity. Furthermore, no N–H stretching at about 3300 cm^−1^ of salim^−^ ligands could be detected in their infrared spectra, which would have been hint of hydrolyzed sal(*p*-X)ben^2−^, and stretching of the iminic C=N bond appeared at 1614 cm^−1^ in all cases. 

The mass spectrum of **6a** in a methanol solution aged for 1 day, instead, completely changed, and new peaks at *m/z* 678, 767 and 821 appeared, assigned to [Fe_2_(sal(*p*-*t*Bu)ben)(salim)(OMe)_2_]^+^, ([Fe_2_(sal(*p*-*t*Bu)ben)(salim)_2_(OMe)]^+^ and [Fe_2_(sal(*p*-*t*Bu)ben)(salim)_2_(OMe)_2_ + Na]^+^, respectively, while the intensity of the peak at *m/z* 911 [M – OMe]^+^, which was the most intense one in the fresh solution, almost disappeared, with a relative intensity of only 5%. Similar results were also obtained with **6e**, and this hints at the hydrolytic tendency of these iron(III) compounds also in the absence of other reagents, such as the base, Equation (5).

[Fe_2_(μ-sal(*p*-X)ben)_2_(μ-OMe)_2_] + H_2_O → [Fe_2_(μ-sal(*p*-X)ben)(μ-OMe)_2_(salim)_2_] + *p*-X-benzaldehyde (5)

In the case of **6b** (Me), **6d** (F), **6f** (CF_3_) and **6g** (NO_2_), after 1 h of stirring the reaction mixture, or in the case of **6c** (H) after a longer reaction time, no or very little solid was left and only deep red solutions were formed. Precipitation upon addition of water or *i*Pr_2_O (followed by washing with water in order to eliminate residual salts) yielded brown-red solids, whose infrared spectra invariably showed the N–H stretching of salim^−^ ligands as a sharp band in the 3296–3316 cm^−1^ range, together with the C=O stretching bands of the released *p*-X-benzaldehyde at 1700–1720 cm^−1^, Equation (5). Surprisingly, in the case of **6c** and **6d**, we detected the same mass peaks at *m/z* 606 and 749, also corresponding to the signals present in the mass spectrum of **2b**. This suggests that, when the hydrolysis takes place on both sal(*p*-X)ben^2−^ ligands, probably accelerated by the addition of water, formation of the stable dinuclear unit [Fe_2_(μ-salmp)(μ-OMe)(salim)_2_] occurs with rearrangement of the salim^−^ fragments to recompose the coordinated salmp^3−^ ligand, promoted by the stabilizing effect of the bridging phenoxido oxygen atom.

### 2.4. Molecular Structures of ***2a***·2AcOEt, ***2a***·2CH_3_CN, ***2b***·1.5H_2_O and ***3c***·2DMF

As reported above, we could obtain crystals suitable for single-crystal X-ray diffraction studies of **2a**·2AcOEt, **2a**·2CH_3_CN and **2b**·1.5H_2_O by slow diffusion of AcOEt, *i*Pr_2_O and again *i*Pr_2_O into the DMF, CH_3_CN and MeOH reaction mixtures, respectively. This was necessary especially for **2a**, which is practically insoluble in all solvents but DMF, as also stated in the literature [34]. The same poor solubility in almost all common solvents was observed for the other four series **3**–**6** with tetradentate ligands, and only in the case of **3b** we obtained a DMF solution with a sufficiently high concentration to allow the crystallization of **3c**·2DMF through liquid diffusion of *i*Pr_2_O, with concomitant OMe ↔ OH scrambling. The strong tendency of these complexes to cocrystallize with several solvent molecules was already reported [23,27] and here was clearly confirmed.

Details of the crystal structure solution and refinement are listed in Table S1 in Supplementary Materials, while selected bond lengths and angles are summarized in Table 2 and Table 3. Data collection was performed at room temperature for all crystals, and structural parameters of **2a**·2CH_3_CN and **2a**·2AcOEt can be compared with both **2a**·2DMF [34] and the manganese(III) derivative **1a**·2CH_3_CN [27] in order to show the changes caused by solvent and metal ion. Comparison of the structure of **2a**·2CH_3_CN at room temperature with the one previously reported at low temperature (153 K) [23] revealed little modification.

**Figure 3:**
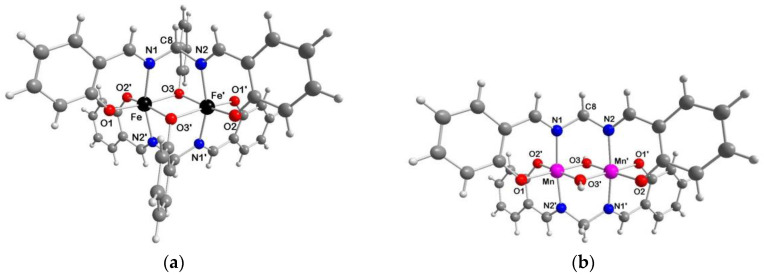
Perspective views of the dinuclear complexes in (**a**) **2a**·2AcOEt and (**b**) **3c**·2DMF with main atom numbering; color code: Fe = black, Mn = violet, O = red, N = blue, C = grey, H = white.

**Figure 4:**
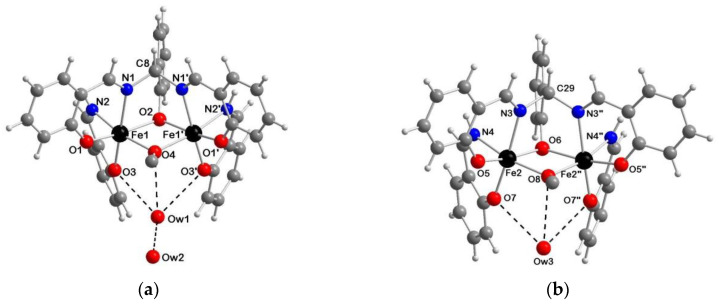
Crystal structures of (**a**) molecule A and (**b**) molecule B of **2b**·1.5H_2_O with main atom numbering; color code: Fe = black, O = red, N = blue, C = grey, H = white, H-bond = dashed black line.

**2a**·2AcOEt, **3c**·2DMF and **2a**·2CH_3_CN (Figure 3 and Figure S2 in Supplementary Materials, respectively) crystallize in the triclinic *P*–1 space group with the complex molecule located about a crystallographic inversion center. In contrast, **2b**·1.5H_2_O (Figure 4) crystallizes in the orthorhombic *Pmc2_1_* space group and the asymmetric unit consists of two independent molecules A and B, located about a crystallographic mirror plane. Besides minor conformational differences of the phenyl groups, the main difference between the two complex molecules is the presence of one (B) or two (A) H-bonded water molecules.

In the crystal structures of **2a**·2AcOEt and **2a**·2CH_3_CN the two iron(III) ions are bridged by two deprotonated pentadentate Schiff base ligands through the iminic nitrogen atoms connected by one carbon atom and the central phenoxido oxygen atom directly bound to both metal centers. Each iron atom is hexa-coordinate (N_2_O_4_) with a pronounced distorted octahedral geometry where, given by the bond distances, the equatorial plane is formed by four oxygen atoms, O1/O2 from the lateral sal moieties and O3/O3’ from the bridging phenoxido group of salmp^3−^, while the elongated axial positions are occupied by the two iminic nitrogen atoms. The greatest deviation from the octahedron is the nonlinearity of the N1–Fe–N2’ system, whose angle is smaller than the ideal value of 180° (161.42(9) and 161.53(7)° for **2a**·2AcOEt and **2a**·2CH_3_CN, respectively). The four equatorial oxygen atoms deviate from the O_4_ least squares (l.s.) plane of about 0.15–0.16 Å in **2a**·2AcOEt and 0.16–0.17 Å in **2a**·2CH_3_CN, while the iron(III) ions resides in this plane (deviations of 0.01 Å, in both cases). In fact, analyzing the structures, it is possible to identify a twist angle between O1FeO2 and O3FeO3’ l.s. planes of 13.0 and 14.3° for **2a**·2AcOEt and **2a**·2CH_3_CN, respectively. The phenoxido bridge forms an angle Fe–O3–Fe = 97.24(9) and 98.32(7)° in the two complexes, with concomitant Fe···Fe distance of 3.071(1) and 3.091(2) Å.

The AcOEt molecules in **2a**·2AcOEt interact with the dinuclear species through two C–H···O H-bonds between the carbonyl group and the central and one iminic C–H units of the salmp^3−^ ligand (C8–H8···O4, *r*_C__···O_ = 3.332(7) Å, CH···O angle = 170.8°; C7–H7···O4, *r*_C__···O_ = 3.553(8) Å, CH···O angle = 156.3°). A picture of the crystal packing is shown in Figure S3 in Supplementary Materials. The CH_3_CN molecules in **2a**·2CH_3_CN, instead, occupy voids within the [Fe_2_(μ-salmp)_2_] units. Comparing the two complexes here described with **2a**·2DMF [34], the latter is more similar to **2a**·2AcOEt regarding both the internal distortion of the octahedral coordination of the iron(III) ions and the solvent interaction. In fact, the DMF molecules also interact with the dinuclear complex through similar C–H···O H-bonds (C8–H13···O4, *r*_C__···O_ = 3.457(9) Å, CH···O angle = 159.5°; C7B–H15B···O4, *r*_C__···O_ = 3.50(1) Å, CH···O angle = 153.2°) as in the case of the AcOEt molecules. The shortest Fe⋯Fe intermolecular distances are 8.309(2), 9.233(2) and 10.179(3) Å for **2a**·2AcOEt, **2a**·2CH_3_CN and **2a**·2DMF, respectively, which reflect the steric and electronic features of the solvent molecules and the different crystal packing in the three crystal structures.

In **2b**·1.5H_2_O, the intact salmp^3−^ binds both iron(III) centers as in **2a**·2solvent, i.e. through the three phenoxido oxygen atoms and the two iminic nitrogen atoms, behaving as pentadentate ligand. The two metal centers are bridged by three different connections: (*i*) the iminic nitrogen atoms, which are connected by one carbon atom, Fe1–N1–C8–N1’–Fe1’ and Fe2–N3–C29–N3”–Fe2” in molecules A and B, respectively (symmetry codes: ‘ = 1–*x*, *y*, *z*; “ = –*x*, *y*, *z*); (*ii*) the central phenoxido oxygen atoms of salmp^3−^, Fe1–O2–Fe1’ and Fe2–O6–Fe2”; (*iii*) the methoxido ions, Fe1–O4–Fe1’ and Fe2–O8–Fe2”. The two salim^−^, instead, derived from the hydrolysis of one salmp^3−^, behave as bidentate ligands for the same iron(III) ion, N2/O3 in molecule A and N4/O7 in molecule B; they invert the coordinative positions so that the nitrogen atom of salmp^3−^ N1/N3 is in trans to the phenoxido oxygen atom of salim^−^ O3/O7. Each iron is hexa-coordinate with a less pronounced octahedral distortion with respect to [Fe_2_(μ-salmp)_2_] (angle N1–Fe1–O3 = 172.6(1), N3–Fe2–O7 = 172.1(1)°). The Fe···Fe distance in this compound is 3.149(1) and 3.150(1) Å in molecules A and B, respectively. Unlike **2a**·2solvent, the l.s. O_3_N equatorial planes in which the two iron ions reside are no longer coplanar, but there is an angle of 19.1(1) (A) and 21.1(1)° (B) between the two. The shortest Fe1⋯Fe2 intermolecular distance is 6.002 Å (Figure S5 in Supplementary Materials).

In the A moiety there are two water molecules OW1 and OW2 that interact via H-bonds with the oxygen atoms of salim^−^ and MeO^−^, with distances OW1···O3 = 2.905(5), OW1···O4 = 3.001(6) and OW1···OW2 = 2.785(11) Å. In B instead only one OW3 water molecule interacts with the dinuclear complex with similar contacts (OW3···O7 = 2.932(6) and OW3···O8= 3.118(8) Å) (Figure 4).

In **3c**·2DMF, each salmen^2−^ acts as tetradentate ligand toward both manganese(III) ions, leading to Mn–N1–C8–N2–Mn’ bridges. In addition, there are two bridging hydroxido groups between the metal ions. The manganese(III) ion is hexa-coordinate (N_2_O_4_) with a slightly distorted octahedral geometry, where the four oxygen atoms with the shortest coordination distances form an almost flat equatorial plane (deviation of atoms of about 0.020 Å from the O_4_ l.s. plane, and manganese resides at 0.015 Å from it), and the highest deviation consists of the nonlinearity of the N1–Mn–N2’ system, 173.20(3)°, smaller than the ideal value (180°). The metal–N/O distances range from 2.0018(7)/ 2.0027(7) Å for Mn–N1/N2’ to 1.9025(7)/1.9123(6) Å for the phenoxido oxygen atoms O1/O2’ and 1.8006(6)/1.7973(6) Å of the hydroxido bridge O3/O3’. These last distances are quite short leading to a very compact Mn_2_O_2_ core where the two manganese(III) ions are very close each other (Mn···Mn’ = 2.6356(6) Å), if compared with **1a**·2CH_3_CN (Mn···Mn’ = 3.111(1) Å) [27]. This is ascribable to the presence of the bridging phenoxido on the central ring in [Mn_2_(μ-salmp)_2_] that imparts further strain to the structure, as also evident by the strong asymmetry of the coordination sites of the two manganese(III) ions. This tension contribution is relieved in **3c**·2DMF with the bridging salmen^2−^ and OH^−^ ligands, and in fact the two coordination sites are similar and, most important, the N1–Mn–N2 angle is closer to 180° (173.20(3)°) than in **1a**·2CH_3_CN (159.1(1)°).

As observed in **2a**·2DMF, the solvent molecule interacts with the dinuclear complex through two C–H···O H-bonds (C8–H8A···O4, *r*_C__···O_ = 3.328(1) Å, CH···O angle = 163.2°; C7–H7···O4, *r*_C__···O_ = 3.286(1) Å, CH···O angle = 160.6°). The overall crystal packing (Figure S4 in Supplementary Materials) leads to the shortest Mn⋯Mn intermolecular distance of 7.957(1) Å.

### 2.5. Cyclic Voltammetry Studies

**Figure 5:**
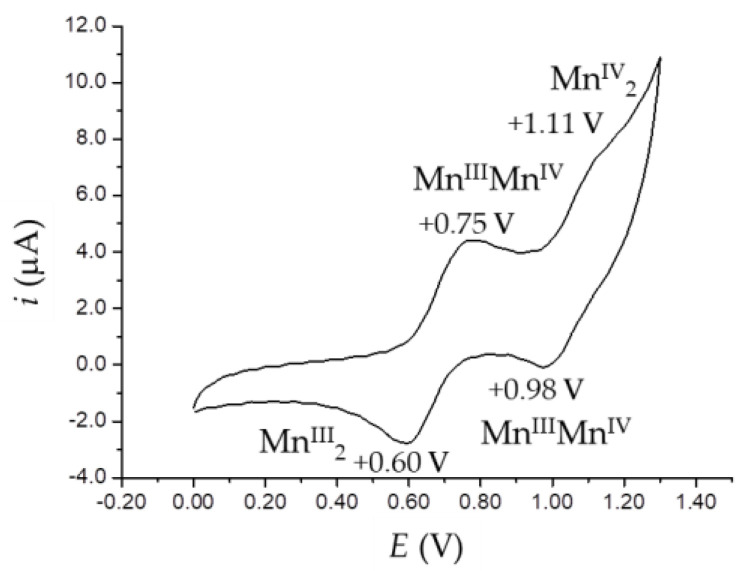
CV scans of **5a** in the oxidation region recorded in DMF 0.1 M TBAPF_6_ at 50 mV/s scan rate; potentials measured vs. Ag/AgCl, 3 M KCl reference electrode.

CV measurements were conducted on four selected derivatives, two of them with manganese, **3b** and **5a**, and two with iron, **4b** and **6a**. Furthermore, **3b** and **4b** have salmen as Schiff base ligands, while **5a** and **6a** have sal(*p*-*t*Bu)ben. Though they are scarcely soluble, their cyclic voltammograms were recorded in DMF solutions with 0.1 M tetrabuthylammonium hexafluorophosphate (TBAPF_6_) as a supporting electrolyte (Figure 5 and Figures S6–S8 in Supplementary Materials) and the obtained electrochemical parameters are summarized in Table 4.

As can be observed, the manganese(III) derivatives were both reduced and oxidized, as reported for [Mn_2_(μ-salmp)_2_] in the literature [27]. In **5a**, two subsequent oxidation steps could be clearly detected at +0.75 and +1.11 V (Figure 5), which imply the formation of the mixed-state species [Mn^III^Mn^IV^(sal(*p*-*t*Bu)ben)_2_(OMe)_2_]^+^ followed by [Mn^IV^_2_(sal(p-*t*Bu)ben)_2_(OMe)_2_]^2+^. Both anodic peaks in the forward scan presented relative cathodic signals in the backward scan, with Δ*E* indicating quasi reversibility of the oxidation processes. In the case of **3b**, instead, one oxidation step at +0.68 V was observed, still fully reversible at +0.63 V (Figure S6 in Supplementary Materials), and this, due to the similarity of the potential with **5a**, seems to suggest the electrochemical formation of [Mn^III^Mn^IV^(salmen)_2_(OMe)_2_]^+^. When moving to the negative region of potentials, **5a** showed two peaks at –0.41 and –0.97 V until the final formation of the Mn^II^_2_, as observed for the iron derivatives, and with no evident backward signals. Interestingly, after exploring the negative potential region, a change of the position of the peaks in the positive potential region was visible shifted to +0.84/+0.56 V (Figure S7 in Supplementary Materials). **3b**, instead, showed two subsequent cathodic peaks at negative potentials, while one of them clearly showing a related anodic signal in the backward scan; in this case the oxidation resulted in not being affected by the negative potential scan (Figure S6 in Supplementary Materials). 

The iron(III) complexes, differently from the manganese(III) ones, were characterized only by the electrochemical reduction to the Fe^II^_2_ species, either in two close but separated steps (**6a**) or in one unique step at a mean potential value (**4b**) (Figure S8 in Supplementary Materials). Compared to [Fe_2_(μ-salmp)_2_] [34], instead, the reduction is irreversible, and this might be attributed to the formation of the neutral species [Fe^II^_2_(μ-salmen)_2_] and [Fe^II^_2_(μ-sal(*p*-*t*Bu)ben)_2_] due to the loss of the methoxido bridging ligands, with a concomitant rearrangement of the coordination environment of the metal ions, which are more stable than those in the anionic [Fe^II^_2_(μ-salmp)_2_]^2−^ derivatives [34], where the iron(II) ions are still trapped by the octahedral embrace of the two bridging pentadentate salmp ligands. 

### 2.6. Magnetic Properties

**Figure 6:**
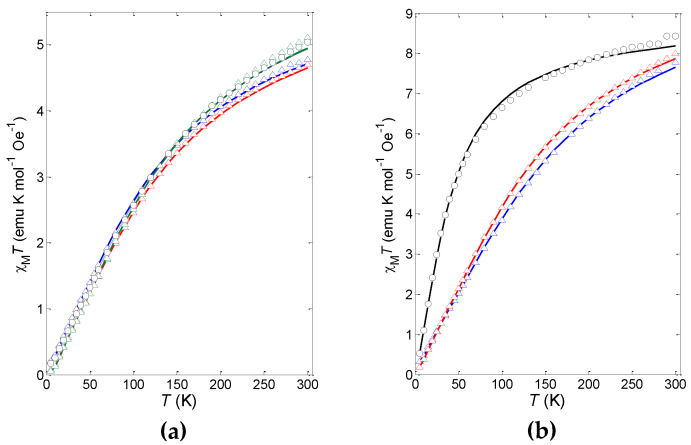
Temperature dependence of the molar susceptibility of (**a**) manganese(III) (**3b**, black circles; **5a**, red triangles; **5e**, blue triangles; **5f**, green triangles) and (**b**) iron(III) (**4b**, black circles; **6a**, red triangles; **6e**, blue triangles) dinuclear compounds; the solid lines are the best-fit curves (see text).

The magnetic susceptibility of selected polycrystalline samples of **3b**, **4b**, **5a**, **5e, 5f**, **6a** and **6e** was measured in the *T* = 5−300 K range under an applied magnetic field of 1 kOe. The molar susceptibility, *χ*_M_, was referred to a dinuclear Mn_2_ or Fe_2_ unit, and data are shown in Figure 6 as temperature dependence of the product χ_M_*T*. The curves of all the analyzed manganese(III) compounds **3b**, **5a**, **5e** and **5f** were very similar, regardless of the Schiff base ligand. As for the iron(III) derivatives, the magnetic behavior of **4b**, bearing the salmen ligand, was quite different from those of **6a** and **6e**, which possessed sal(*p*-X)ben ligands (see Figure S9 in Supplementary Materials for individual plots).

For the iron(III) complexes, χ_M_*T* at 300 K was about 8 emu K mol^−1^ Oe^−1^, which was slightly lower than the expected value for two uncoupled iron(III) ions (8.77 emu K mol^−1^ Oe^−1^, *g* = 2.00). It then decreased upon cooling, reaching values close to zero at 5 K in all cases. Such behavior suggests that the iron(III) ions are antiferromagnetically (AFM) coupled in the dinuclear species, which have an *S* = 0 ground state. The χ_M_*T* decrease of **6a** and **6e** was already evident at room temperature, while that of **4b** occurred at a lower temperature, thus suggesting a weaker exchange coupling in **4b** with respect to **6a** and **6e**. For the manganese(III) derivatives **3b**, **5a**, **5e** and **5f**, at 300 K χ_M_*T* ≈ 5 emu K mol^−1^ Oe^−1^, lower than the expected value for two uncoupled manganese(III) ions (6.02 emu K mol^−1^ Oe^−1^, *g* = 2.00). Then the χ_M_*T* product quickly decreased upon cooling, suggesting that the manganese(III) ions strongly interacted through AFM super-exchange coupling in all compounds. 

The fitting of *χ*_M_ of polynuclear magnetic compounds was a nontrivial issue, especially for manganese [39], in view of the overparameterization problem. We then decided to use an in-house software, based on full Hamiltonian diagonalization and Levenberg-Marquardt optimization, by first fitting χ_M_*T* data to the sum of two terms: (*i*) a Hamiltonian containing the isotropic exchange and Zeeman terms for the coupled dinuclear species, as reported in Equation (6), and (*ii*) the Zeeman term for the molar uncoupled fraction *f* [40], further augmented with a TIP component fixed at 600 × 10^−6^ emu mol^−1^, value typical for manganese(III) ions [41]. The best-fit parameters revealed to be relatively insensitive to the TIP value.

*Ĥ* = −2*J***Ŝ**_1_**·Ŝ**_2_ + *g* μ_B_ (**Ŝ**_1_+**Ŝ**_2_) *B* (6)

Addition of the zero-field splitting and mean-field inter-dinuclear interaction to the Hamiltonian did not improve the fit quality nor significantly change the best fit values of the *J*, *g* and *f* parameters. The obtained results were confirmed by independently fitting the χ_M_*T* data using the PHI 3.1.5 software [42]. The best fit *J*, *g* and *f* parameters are collected in Table 5. The fraction of uncoupled impurity *f* ranged from 0.8% in **5f** to 3.4% in **5e**, which can be considered acceptable for these poorly soluble complexes.

All *J* constants are negative, showing that the metal-metal super-exchange coupling is AFM. Despite the significant variation going from **4b** to **6a** and **6e**, the values fall in the −2.2 to −16.4 cm^−1^ range expected for *bis*(μ-alkoxido)diiron(III) complexes [23]. On the other hand, the two **2a**·solvent derivatives are reported to possess a weak ferromagnetic (FM) coupling between the two iron(III) ions, in particular **2a**·2DMF [34]: *J* = +1.21 cm^− 1^, *zJ*’ = –0.018 cm^−1^ (*g* probably assumed equal to 2.00 by the Authors) and Fe–O–Fe = 97.06(9)°; **2a**·2CH_3_CN [23]: *J* = +0.48(1) cm^−1^, *g* = 2.02(1), *zJ′* = −0.03(1) cm^−1^, TIP = 0.0016(2) cm^3^ mol^−1^ and Fe–O–Fe = 98.01(6)°. It is immediately clear that salmen^2−^ and sal(*p*-X)ben^2−^ ligands, even if strictly related to salmp^3−^, produce a super-exchange Fe–O(Me)–Fe pathway with a wider angle, probably caused by the absence of the bridging phenoxido atom on the central ring. Taking into account the suggested magneto-structural relationship [23], the Fe–O–Fe angle should be close to 101° for **4b** and around 103° for **6a** and **6e**, even if this remains just a speculation in the absence of their crystal structures. Regardless, the small difference between **6a** (X = *t*Bu) and **6e** (X = Cl) is most probably not related to the substituent X, which is far and in an unfavorable position for inducing any electronic effect on the coupling constant, differently from what we observed in trinuclear copper(II) hydroxido propellers with electronically-modulated tridentate Schiff base ligands [43].

In **4b** the AFM coupling between the iron(III) centers is much weaker that in the manganese(III) counterpart **3b**, while the difference is less marked when comparing [M_2_(μ-sal(*p*-X)ben)_2_(μ-OMe)_2_] complexes, where the manganese(III) derivatives are slightly more AFM coupled compared to the iron(III) ones. **3b**, **5a**, **5e** and **5f** displayed coupling constants *J* in the range −12.3 to −13.6 cm^−1^, which, to the best of our knowledge, are the most negative (AFM) values reported so far. 

Most manganese(III) dinuclear compounds feature FM coupling, especially those classified as out-of-plane dimers [39]. In these complexes, the plane defined by the N and O donor atoms of the Schiff base ligands is perpendicular to the plane defined by the two Mn–O–Mn bridges, leading to alignment of the individual Jahn–Teller axes. For instance, the out-of-plane [Mn(salmen)(μ-N_3_)]_2_ complex (salmen^2−^ ligand is in this case a salen ligand with a methyl group on the ethylene bridge) has *J* = +0.58 or +0.85 cm^−1^, when either the ZFS or inter-dimer *zJ*’ interaction, respectively, is included in the fit [44]. The rare AFM-coupled out-of-plane dimers have *J* in the range −0.45 to −3.25 cm^−1^ but the significance of these values has been questioned [39]. On the other hand, **1a**·2DMF is reported to have *J* = −3.3(2) cm^−1^ and *g* = 1.96(8) [27], getting closer to the values observed in **3b**, **5a**, **5e** and **5f**.

The family of dimeric species [Mn_2_O(R-sao)(tpa)_2_](ClO_4_)]_2_ (R-saoH_2_ = R-salicylaldoxime, with R = H, Et and Ph, and tpa = *tris*(2-pyridylmethyl)amine) have been recently reported, where the manganese(III) ions are linked by a Mn–N–O–Mn oxime and an Mn–O–Mn oxido bridge leading to AFM coupling with *J* = −4.94, −3.87, −5.17 cm^−1^ for R = H, Et, Ph, respectively [45]. The related complexes (Mn_2_(R-sao)_2_(dpa)_2_)(ClO_4_)_2_ (R = H, Me, Et and Ph, and dpa = di(2-picolyl)amine), having two Mn–N–O–Mn oxime bridges, display AFM coupling with *J* = −5.73, −3.63, −5.63, –2.05 cm^−1^ for R = H, Me. Et, Ph, respectively [46]. According to DFT calculations, these complexes can be classified as out-of-plane dimers since the manganese(III) Jahn–Teller axes are parallel. However, we note that in these complexes the core is a six-membered ring with chair conformation instead of the Mn_2_O_2_ planar core of conventional out-of-plane dimeric species. 

The coordination pattern in the present manganese(III) dinuclear complexes substantially differ from that of previously reported examples in that each Schiff base coordinates both metal ions instead of a single one, realizing an unusual N_2_O_4_ pattern with *trans* N atoms and two *trans* Mn–O(Me)–Mn bridges. This pattern does not in principle prevent that our complexes are out-of-plane dimers. However, the short bridge between the imino N atoms (*i*) causes a distortion of the N–Mn–N axis (N–Mn–N angle < 180°) and (*ii*) may provide an exchange pathway additional to the methoxido bridges. On the other hand, as outlined in the structural section, the absence of the central phenoxido bridging unit on going from **1a** to **3c**, but also to **3b**, **5a**, **5e** and **5f**, relieves part of the internal strain allowing closer manganese(III) ions, shorter Mn–O distances and smaller Mn–O–Mn angles, which are probably responsible for the stronger AFM coupling. As also discussed in the case of the iron(III) derivatives, especially in these manganese(III) complexes the small differences between **3b**, **5a** (X = *t*Bu), **5e** (Cl) and **5f** (CF_3_) are not related to the substituent X, especially considering that the central aromatic ring is completely absent in **3b**.

## 3. Materials and Methods 

### 3.1. General Procedures

All used chemicals were reagent grade. Solvents were used as received. Elemental analyses were performed at the Microanalytical Laboratory at the Università degli Studi di Milano. ESI-MS spectra were recorded on MeOH or MeCN solutions with an LCQ Advantage Thermofluxional instrument. Infrared spectra were recorded using a JASCO FT-IR 410 spectrophotometer as KBr disks with a 2 cm^−1^ resolution. H_3_salmp [2], H_2_salmen [5] and H_2_sal(*p*-X)ben (X = *t*Bu, Me, H, F, Cl, CF_3_, NO_2_) [4,11,19,20,31] were synthesised following the synthetic procedure reported in the literature.

### 3.2. Synthesis of [Mn_2_(μ-salmp)_2_] (***1a***)

This compound was obtained by the modification of a literature procedure [27]. Solid Mn(AcO)_3_·2H_2_O (110.2 mg, 0.41 mmol) was added to a yellow solution of H_3_salmp (142.4 mg, 0.41 mmol) in MeOH (15 mL) and Et_3_N (1.5 mL) and the mixture was stirred at room temperature for 6 h. The dark brown solid formed was filtered, washed with MeOH, *i*Pr_2_O and dried under vacuum. Yield: 113.9 mg (65%). Elemental analysis calcd (%) for C_42_H_30_Mn_2_N_4_O_6_·2H_2_O (832.62): C 60.59, H 4.12, N 6.73. Found: C 60.66, H 4.36, N 6.52. MS-ESI^+^ (MeOH): *m/z* 797 ([M + H]^+^, 20%), 819 ([M + Na]^+^, 100), 1614 ([2M + Na]^+^, 40). IR (KBr): 1608 (*ν* C=N) cm^−1^. The synthesis was also conducted in DMF as solvent under similar conditions, obtaining a very dark solid, washed with DMF and *i*Pr_2_O and dried under vacuum. Yield: 22%. Elemental analysis calcd (%) for C_42_H_30_Mn_2_N_4_O_6_·1.5DMF (927.76): C 60.19, H 4.40, N 8.30. Found: C 60.20, H 4.19, N 8.44. IR (KBr): 1663 (*ν* C=O_DMF_), 1608 (*ν* C=N) cm^−1^.

### 3.3. Synthesis of [Fe_2_(μ-salmp)_2_] (***2a***)

This compound was obtained by modification of a literature procedures [3,8,23,28]. Solid FeCl_3_ (168.8 mg, 1.04 mmol) was added to a solution of H_3_salmp (358.2 mg, 1.03 mmol) in MeOH (15 mL) and Et_3_N (1.5 mL) and the mixture was stirred at room temperature for 6 h. The brown-red solid formed was filtered, washed with MeOH, *i*Pr_2_O and dried under vacuum. Yield: 370.2 mg (86%). Elemental analysis calcd (%) for C_42_H_30_Fe_2_N_4_O_6_·2H_2_O (834.45): C 60.45, H 4.11, N 6.71. Found: C 60.69, H 4.35, N 6.81. MS-ESI^+^ (MeOH): *m/z* 821 ([M + Na]^+^, 100%), 1618 ([2M + Na]^+^, 50). IR (KBr): 1609 (*ν* C=N) cm^−1^. The synthesis was also conducted in CH_3_CN or DMF as solvent under similar conditions, with final lower yields for co-precipitation of Et_3_NHCl in the former case, which needed extensive washing with a H_2_O:MeOH 1:1 solvent mixture (yield: 37%), or high solubility in the latter one (yield: 25%), as also observed in the case of the manganese(III) derivative **1a**. Elemental analysis calcd (%) for C_42_H_30_Fe_2_N_4_O_6_·1.5DMF·5H_2_O (998.13): C 54.41, H 5.64, N 8.71. Found: C 54.50, H 5.52, N 8.90. IR (KBr): 1668 (*ν* C=O DMF), 1610 (*ν* C=N) cm^−1^. Single crystals suitable for X-ray diffraction were obtained by slow diffusion either of AcOEt into the DMF reaction mixture (**2a**·2AcOEt) or *i*Pr_2_O into the MeCN reaction mixture (**2a**·2MeCN). Substitution of FeCl_3_ with Fe(NO_3_)_3_·9H_2_O yielded similar results.

### 3.4. Synthesis of [Fe_2_(μ-salmp)(μ-OMe)(salim)_2_] (***2b***)

Solid FeCl_3_ (146.4 mg, 0.90 mmol) was added to a solution of H_3_salmp (314.4 mg, 0.91 mmol) in MeOH (15 mL) and NaOH (4.1 mL of a 0.66 mol L^−1^ aqueous solution, 2.71 mmol) and the mixture was stirred at room temperature for 6 h. The formed vivid red solid was filtered, washed with MeOH and *i*Pr_2_O and dried under vacuum. Yield: 185.4 mg (50%). Elemental analysis calcd (%) for C_36_H_30_Fe_2_N_4_O_6_·1.5H_2_O·2MeOH (817.45): C 55.83, H 5.06, N 6.85. Found: C 55.57, H 4.50, N 6.74. MS-ESI^+^ (MeOH): *m/z* 606 ([M – salim]^+^, 80%), 749 ([M + Na]^+^, 100). IR (KBr): 3302 (ν N–H salim), 1615 (*ν* C=N) cm^−1^. Single crystals of **2b**·1.5H_2_O suitable for X-ray diffraction were obtained from slow diffusion of *i*Pr_2_O into the methanolic reaction mixture.

### 3.5. Synthesis of [Fe_2_(μ-salmp)(μ-OH)(salim)_2_] (***2c***)

Solid FeCl_3_ (158.6 mg, 0.98 mmol) was added to a solution of H_3_salmp (313.2 mg, 0.90 mmol) in DMF (20 mL) and NaOH (4.4 mL of a 0.66 mol L^−1^ aqueous solution, 2.70 mmol) and the mixture was stirred at room temperature for 6 h. The formed dark red solid was filtered, washed with DMF and *i*Pr_2_O and dried under vacuum. Yield: 285.6 mg (70%). Elemental analysis calcd (%) for C_35_H_28_Fe_2_N_4_O_6_·1.5DMF·H_2_O (839.98): C 57.16, H 5.36, N 9.15. Found: C 56.73, H 5.20, N 9.14. MS-ESI^+^ (MeOH): *m/z* 605 ([M – salim – OH + OMe]^+^, 100%), 749 ([M – OH + OMe + Na]^+^, 30) (OH/OMe exchange in MeOH solution). IR (KBr): 3302 (*ν* N–H salim), 1660 (*ν* C=O DMF), 1615 (*ν* C=N) cm^−1^.

### 3.6. Synthesis of [Mn_2_(μ-salmen)_2_(μ-OEt)_2_] (***3a***)

Solid Mn(AcO)_3_·2H_2_O (54.1 mg, 0.20 mmol) was added to a solution of H_2_salmen (50.2 mg, 0.20 mmol) in EtOH (15 mL) and Et_3_N (3 mL) and the mixture was left under stirring at room temperature for 6 h. The obtained dark green solid was filtered, washed with EtOH and *i*Pr_2_O and dried under vacuum. Yield: 52.3 mg (75%). Elemental analysis calcd (%) for C_34_H_34_Mn_2_N_4_O_6_ (704.53): C 57.96, H 4.86, N 7.95. Found: C 58.37, H 4.77, N 8.36. MS-ESI^+^ (MeOH): *m/z* 645 ([M – 2OEt + OMe]^+^, 100%), 727 [M + Na]^+^, 10) (OEt/OMe exchange in MeOH solution). IR (KBr): 1623 (*ν* C=N) cm^−1^. For the synthesis of **3b** and **3c** see Supplementary Materials.

### 3.7. Synthesis of [Fe_2_(μ-salmen)_2_(μ-OEt)_2_] (***4a***)

Solid FeCl_3_ (32.6 mg, 0.20 mmol) and Et_3_N (3 mL) were added to a solution of H_2_salmen (50.4 mg, 0.20 mmol) in EtOH (15 mL) and the mixture was left under stirring at room temperature for 6 h. The obtained dark brown solid was filtered, washed with EtOH, *i*Pr_2_O and dried under vacuum. Yield: 37.1 mg (53%). Elemental analysis calcd (%) for C_34_H_34_Fe_2_N_4_O_6_ (706.39): C 57.81, H 4.85, N 7.93. Found: C 57.50, H 4.71, N 8.13. MS-ESI^+^ (MeOH): *m/z* 647 ([M – 2OEt + OMe]^+^, 100%), 701 ([M – 2OEt + 2OMe + Na]^+^, 75), 729 [M + Na]^+^, 20) (OEt/OMe exchange in MeOH solution). IR (KBr): 1618 (*ν* C=N) cm^−1^. For the synthesis of **4b** and **4c** see Supplementary Materials.

### 3.8. Synthesis of [Mn_2_(μ-sal(p-tBu)ben)_2_(μ-OMe)_2_] (***5a***)

Solid Mn(AcO)_3_·2H_2_O (71.9 mg, 0.42 mmol) was added to a yellow suspension of H_2_sal(*p*-*t*Bu)ben (155.5 mg, 0.40 mmol) in MeOH (3 mL) with a colour change to brown-green. Et_3_N (1.5 mL) was added yielding to the precipitation of a dark brown solid. The reaction mixture was left under stirring for 4 h at room temperature, and then the solid was filtered, washed with MeOH and *i*Pr_2_O and dried under vacuum. Yield: 78.9 mg (41%). Elemental analysis calcd (%) for C_52_H_54_Mn_2_N_4_O_6_·1/3H_2_O (946.91): C 65.95, H 5.86, N 5.92. Found: C 65.95, H 5.85, N 5.77. MS-ESI^+^ (MeOH): *m/z* 909 ([M – OMe]^+^, 100%), 941 ([M + H]^+^, 70). IR (KBr): 1621 (*ν* C=N) cm^−1^. For the synthesis of **5b**–**g** see Supplementary Materials.

### 3.9. Synthesis of [Fe_2_(μ-sal(p-tBu)ben)_2_(μ-OMe)_2_] (***6a***)

Solid FeCl_3_ (88.7 mg, 0.55 mmol) was added to a suspension of H_2_sal(*p*-*t*Bu)ben (167.0 mg, 0.43 mmol) in MeOH (3 mL) and then Et_3_N (1.5 mL) was added, with a concomitant colour change from blue/violet to dark red and the formation of a precipitate. The reaction mixture was then left under stirring at room temperature for 1 h, after that the solid was collected by filtration, washed with MeOH and *i*Pr_2_O and dried under vacuum. Yield: 145.1 mg (65%). Elemental analysis calcd (%) for C_52_H_54_Fe_2_N_4_O_6_·2.5H_2_O (987.76): C 63.23, H 6.02, N 5.67. Found: C 63.30, H 6.23, N 5.64. IR (KBr): 1614 (*ν* C=N) cm^−1^. MS-ESI^+^ (MeOH) on freshly-prepared solution: *m/z* 911 ([M – OMe]^+^, 100%), 965 ([M + Na]^+^, 40). MS-ESI^+^ (MeOH) on aged solution for 1 day: *m/z* 678 ([Fe_2_(sal(*p*-*t*Bu)ben)(salim)(OMe)_2_]^+^, 20%), 767 ([Fe_2_(sal(*p*-*t*Bu)ben)(salim)_2_(OMe)]^+^, 15), 821 ([Fe_2_(sal(*p*-*t*Bu)ben)(salim)_2_(OMe)_2_ + Na]^+^, 100), 911 ([M – OMe]^+^, 5). Substitution of FeCl_3_ with Fe(NO_3_)_3_·9H_2_O yielded similar results. For the synthesis of **6b**–**g** see Supplementary Materials.

### 3.10. X-ray Data Collection and Structure Determination

Crystals of H_2_sal(*p*-*t*Bu)ben and H_2_sal(*p*-CF_3_)ben suitable for X-ray diffraction studies were obtained from slow evaporation of chloroform solutions, crystals of **2a**·2AcOEt were obtained from slow diffusion of AcOEt into DMF, crystals of **2a**·2CH_3_CN were obtained from slow diffusion of *i*Pr_2_O into CH_3_CN, crystals of **2b**·1.5H_2_O were obtained from slow diffusion of *i*Pr_2_O into MeOH, while crystals of **3c**·2DMF were obtained by slow diffusion of *i*Pr_2_O into a DMF solution of **3b**. Intensity data were collected at room temperature on a Bruker Apex II CCD diffractometer, using graphite-monochromatized Mo-K*α* radiation (*λ* = 0.71073 Å). Intensity data were corrected for Lorentz-polarization effects and for absorption (SADABS [47]). The structures were solved by direct methods (SIR-97 [48]) and completed by iterative cycles of full-matrix least squares refinement on *F*_o_^2^ and *ΔF* synthesis using SHELXL program [49] (WinGX suite [50]). Hydrogen atoms, located on the ∆*F* maps, were allowed to ride on their carbon atoms. A summary of the crystal data and refinement details for all compounds is given in Table S1 in Supplementary Materials.

CCDC 2030845–2030850 contains the supplementary crystallographic data for **2a**·2CH_3_CN, H_2_sal(*p*-CF_3_)ben, **3c**·2DMF, H_2_sal(*p*-*t*Bu)ben, **2b**·1.5H_2_O and **2a**·2AcOEt, respectively. These data can be obtained free of charge via http://www.ccdc.cam.ac.uk/conts/retrieving.html, or from the Cambridge Crystallographic Data Centre, 12 Union Road, Cambridge CB2 1EZ, UK; fax: (+44) 1223-336-033, or e-mail: deposit@ccdc.cam.ac.uk.

### 3.11. Cyclic Voltammetry

Electrochemical tests were carried out with an Autolab PGSTAT 12 electrochemical instrument (Ecochemie). The experiments were performed in a single-compartment three-electrode cell at room temperature under Ar atmosphere. Pt was used as working electrode, a glassy carbon rod served as auxiliary electrode and an aqueous Ag/AgCl, KCl 3 M electrode (Metrohm) was the reference electrode; all the potential values given are referred to such an electrode. The solutions were prepared dissolving a small amount of **3b**, **4b**, **5a** and **6a** in a solution of DMF containing 0.1 M TBAPF_6_ as supporting electrolyte.

### 3.12. Magnetic Measurements

The magnetic moment *μ* of powder samples of **3b**, **4b**, **5a**, **5e, 5f**, **6a**, and **6e** was measured between 5 and 300 K using a Quantum Design MPMS XL-5 SQUID magnetometer. Weighed amounts (about 15 mg) of the compounds were sealed in polycarbonate capsules and *μ* was measured under an applied magnetic field of 1 kOe from 300 K down to 5 K. The molar susceptibility was obtained as *χ*_M_ = (*μ*/*H*) × (MW/*m*), where MW is the molecular weight of the complex, *m* is the sample mass, and *H* is the applied magnetic field. Diamagnetic contributions were subtracted from *μ* before calculating *χ*_M_. The ligand diamagnetism was estimated using Pascal’s constants [51].

## 4. Conclusions

The present paper aimed to show a new piece of chemistry, i.e. the reactivity of the shortened salen-type analogues H_3_salmp, H_2_salmen and H_2_sal(*p*-X)ben (X = *t*Bu, Me, H, F, Cl, CF_3_, NO_2_) with manganese(III) and iron(III), which invariably formed dinuclear complexes with bridging Schiff base ligands and alkoxido ions of general formula [M_2_(μ-salmp)_2_], [M_2_(μ-salmen)_2_(μ-OR)_2_] and [M_2_(μ-sal(*p*-X)ben)_2_(μ-OMe)_2_] (M = Mn, Fe; R = H, Me, Et). Complexes of iron(III) with sal(*p*-X)ben^2−^ showed the tendency to hydrolyse to salim^−^ in solution and in some cases rearrange to salmp^3−^, while complexes of manganese(III) are more stable with no hints of C–N bond breakage. A similar ligand rupture was evidenced by reaction of H_3_salmp with iron(III) salts in the presence of NaOH as a stronger base than NEt_3_, leading to the dinuclear compound [Fe_2_(μ-salmp)_2_(μ-OMe)(salim)_2_], where part of the tension created by two salmp^3−^ bridging ligands in [Fe_2_(μ-salmp)_2_] is relieved by one methoxido bridging ion. Infrared spectroscopy, mass spectrometry and structural characterization by single-crystal X-ray diffraction were used in combination to follow the reactivity.

CV measurements highlight the possibility to modulate the oxidation state of the complexes analysed with the formation of mixed-state Mn^III^Mn^IV^ and Mn^II^Mn^III^ species. The electrochemical behaviour of the iron(III) derivatives, which reveal to be irreversibly reduced and suggest the formation of stable [Fe^II^_2_(μ-L)_2_] complexes, is peculiar and certainly aim of our future efforts. Magnetic studies showed the presence of AFM super-exchange among the two metal ions within selected dinuclear complexes, with negligible effect of the substituent X, too far and in an unfavorable position for inducing any electronic effect on the coupling constants, which are then dominated by the structural distortion caused by the shortened salen-type ligands. In particular, the manganese(III) complexes show, to the best of our knowledge, the strongest AFM coupling reported so far, and this consolidates the usefulness of these ligands in different fields and the profit doing research with them. 

Very little is known but so much needs to be discovered on these shortened Schiff bases, their behavior as ligands and the properties of the related metal complexes, and as the Italian poet Giacomo Leopardi wrote at the end of his famous poem ‘L’infinito’ [52] (The infinity), ‘e il naufragar m’è dolce in questo mar’ (and foundering in this sea is sweet to me), a sea of exciting chemistry.

## Figures and Tables

**Table 1:** Formulae and codes of dinuclear compounds object of study in this paper.

Formula	Compound Code
[M_2_(μ-salmp)_2_]	**1a** (M = Mn), **2a** (M = Fe)
[Fe_2_(μ-salmp)(μ-OR)(salim)_2_]	**2b**–**c** (R = Me, H)
[Mn_2_(μ-salmen)_2_(μ-OR)_2_]	**3a**–**c** (R = Et, Me, H)
[Fe_2_(μ-salmen)_2_(μ-OR)_2_]	**4a**–**c** (R = Et, Me, H)
[Mn_2_(μ-sal(*p*-X)ben)_2_(μ-OMe)_2_]	**5a**–**g** (X = *t*Bu, Me, H, F, Cl, CF_3_, NO_2_)
[Fe_2_(μ-sal(*p*-X)ben)_2_(μ-OMe)_2_]	**6a**–**g** (X = *t*Bu, Me, H, F, Cl, CF_3_, NO_2_) ^1^

^1^ only **6a**, **6c** and **6e** could be isolated in the solid state.

**Table 2:** Selected bond distances (Å) and angles (°) of **2a**·2AcOEt, **2a**·2CH_3_CN, **2a**·2DMF [34], **1a**·2CH_3_CN [27] and **3c**·2DMF, all collected at room temperature ^1,2^.

Parameter	2a·2AcOEt ^3^	2a·2CH_3_CN ^3^	2a·2DMF ^3^	1a·2CH_3_CN ^4^	3c·2DMF ^4^
M–O1	1.897(2)	1.896(2)	1.894(3)	1.857(3)	1.9025(7)
M–O2’	1.917(2)	1.905(2)	1.921(3)	1.952(3)	1.9123(6)
M–N1	2.142(2)	2.164(2)	2.156(3)	2.088(3)	2.0018(7)
M–N2’	2.149(2)	2.144(2)	2.138(3)	2.196(3)	2.0027(7)
M–O3	2.033(2)	2.028(2)	2.023(2)	2.239(3)	1.8006(6)
M–O3’	2.059(2)	2.057(2)	2.064(2)	1.900(3)	1.7973(6)
M···M’	3.071(1)	3.091(2)	3.063(1)	3.111(1)	2.6357(4)
M–O3–M’	97.24(9)	98.32(7)	97.06(9)	97.1(1)	94.20(2)
O3–M–O3’	82.76(9)	81.68(7)	82.94(9)	82.9(1)	85.80(2)
O1–M–O2’	94.17(12)	94.24(8)	93.6(1)	93.3(1)	88.79(3)
O2’–M–O3	91.78(10)	92.07(8)	91.6(1)	93.0(1)	91.89(3)
O1–M–O3’	92.68(10)	93.69(7)	93.4(1)	91.0(1)	93.54(3)
O2’–M–N2’	87.04(10)	86.97(7)	88.3(1)	86.5(1)	90.82(3)
O2’–M–N1	107.03(10)	107.62(8)	101.3(1)	113.2(1)	95.02(3)
O1–M–N2’	102.91(9)	102.64(7)	108.4(1)	95.4(2)	91.82(3)
O1–M–N1	88.42(10)	87.86(7)	87.0(1)	90.9(1)	91.78(3)
O3–M–N2’	85.07(9)	86.42(7)	86.9(1)	79.9(1)	88.50(3)
O3–M–N1	82.47(9)	81.89(7)	82.5(1)	80.1(1)	87.83(3)
O3’–M–N2’	82.37(9)	81.75(7)	81.8(1)	85.2(1)	86.87(3)
O3’–M–N1	82.44(9)	82.44(7)	81.8(1)	86.4(1)	87.15(3)
O2’–M–O3’	168.44(9)	167.40(6)	170.5(1)	166.0(1)	176.76(3)
O1–M–O3	170.27(9)	169.21(6)	167.8(1)	173.6(1)	179.25(3)
N1–M–N2’	161.42(9)	161.53(7)	161.4(1)	159.1(1)	173.20(3)

^1^ Primed atoms are related to the unprimed atoms by an inversion center; ^2^ literature data were adapted in order to maintain the same atom numbering applied in this work; ^3^ M = Fe; ^4^ M = Mn.

**Table 3:** Selected bond distances (Å) and angles (°) of **2b**·1.5H_2_O ^1^.

Molecule A	Value	Molecule B	Value
Fe1–O1	1.917(3)	Fe2–O5	1.908(4)
Fe1–O2	2.069(3)	Fe2–O6	2.065(3)
Fe1–O3	1.940(3)	Fe2–O7	1.935(4)
Fe1–O4	1.985(2)	Fe2–O8	1.990(3)
Fe1–N1	2.175(3)	Fe2–N3	2.163(4)
Fe1–N2	2.086(4)	Fe2–N4	2.081(4)
Fe1···Fe1’ ^1^	3.149(1)	Fe2···Fe2” ^2^	3.151(1)
Fe1–O2–Fe1’ ^1^	99.1(2)	Fe2–O6–Fe2” ^2^	99.4(2)
Fe1–O4–Fe1’ ^1^	105.0(2)	Fe2–O8–Fe2” ^2^	104.7(2)
O1–Fe1–N2	95.05(15)	O5–Fe2–N4	93.42(18)
N2–Fe1–O2	89.20(14)	N4–Fe2–O6	87.43(17)
O2–Fe1–O4	77.17(12)	O6–Fe2–O8	77.20(15)
O4–Fe1–O1	98.27(15)	O8–Fe2–O5	101.20(18)
O1–Fe1–N1	86.93(14)	O5–Fe2–N3	86.42(16)
O1–Fe1–O3	98.41(13)	O5–Fe2–O7	97.23(15)
N2–Fe1–N1	88.33(15)	N4–Fe2–N3	85.93(19)
N2–Fe1–O3	86.05(14)	N4–Fe2–O7	86.84(18)
O2–Fe1–N1	83.15(15)	O6–Fe2–N3	83.68(19)
O2–Fe1–O3	91.89(15)	O6–Fe2–O7	92.79(19)
O4–Fe1–N1	89.41(15)	O8–Fe2–N3	89.42(18)
O4–Fe1–O3	94.91(15)	O8–Fe2–O7	96.72(18)
O1–Fe1–O2	169.10(15)	O5–Fe2–O6	169.98(19)
N2–Fe1–O4	166.35(14)	N4–Fe2–O8	164.35(17)
N1–Fe1–O3	172.56(14)	N3–Fe2–O7	172.08(18)

^1^ symmetry code: 1–*x*, *y*, *z*; ^2^ symmetry code: –*x*, *y*, *z*

**Table 4:** Electrochemical data (V) for **3b**, **4b**, **5a** and **6a** recorded in DMF 0.1 M TBAPF_6_ at 50 mV/s scan rate; potentials measured vs. Ag/AgCl, 3 M KCl reference electrode.

Compound	Redox Couple	*E*_pa_	*E*_pc_	Δ*E*	*E*_½_
**3b**	Mn^IV^/Mn^III^	+0.68	+0.63	0.05	+0.66
	Mn^III^/Mn^II^	−0.00	−0.30	0.30	−0.15
		– ^1^	−0.90	–	–
**4b**	Fe^III^/Fe^II^	– ^1^	−0.77	–	–
**5a**	Mn^IV^/Mn^III^	+0.75	+0.60	0.15	+0.68
		+1.11	+0.98	0.13	+1.05
	Mn^III^/Mn^II^	– ^1^	−0.41	–	–
		– ^1^	−0.97	–	–
**6a**	Fe^III^/Fe^II^	– ^1^	−0.70	–	–
		– ^1^	−0.84	–	–

^1^ irreversible.

**Table 5:** Best fit values of the magnetic coupling constants *J*, *g* factors and uncoupled fractions *f* for **3b**, **4b**, **5a**, **5e**, **5f**, **6a** and **6e**.

Compound	*J* (cm^−1^)	*g* ^1^	*f* ^2^
**3b**	–13.64(6)	2.05	2.8%
**4b**	–2.88(2)	2.02	1.0%
**5a**	–12.95(4)	1.98	1.2%
**5e**	–12.25(5)	1.97	3.4%
**5f**	–13.61(8)	2.07	0.8%
**6a**	–9.517(8)	2.20	1.6%
**6e**	–10.71(3)	2.21	2.9%

^1^ The estimated accuracy of *g* values is ± 0.01; ^2^ the estimated accuracy of the fractions *f* is ±0.1%.

## Supplementary Materials

Supplementary materials can be found at https://www.mdpi.com/1422-0067/21/21/7882/s1. Experimental Section (cont.), crystallographic data for H_2_sal(*p*-*^t^*Bu)ben, H_2_sal(*p*-CF_3_)ben, 2a·2AcOEt, 2a·2CH_3_CN, 2b·1.5H_2_O and 3c·2DMF (Table S1); crystal packing of H_2_sal(CF_3_)ben (Figure S1); crystal structure and crystal packing of 2a·2CH_3_CN (Figure S2); crystal packing of 2a·2AcOEt (Figure S3); crystal packing of 3c·2DMF (Figure S4); crystal packing of 2b·1.5H_2_O (Figure S5); CV scans of 3b, 4b, 5a and 6a recorded in DMF 0.1 M TBAPF_6_ at 50 mV/s scan rate (Figures S6–S8); temperature dependence of the molar susceptibility of manganese(III) (3b, 5a, 5e, 5f) and iron(III) (4b, 6a, 6e) dinuclear compounds (Figure S9).

## Abbreviations

H_3_salmp	*N,N*′-*bis*(salicylidene)-2-hydroxyphenylmethanediamine
H_2_salmen	*N,N*′-*bis*(salicylidene)methanediamine
H_2_sal(*p*-X)ben	*N,N*′-*bis*(salicylidene)-4-X-phenylmethanediamine
Hsalim	salicylaldimine
salH	salicylaldehyde
DMF	dimethylformamide
AcOEt	ethyl acetate
RT	room temperature
MS-ESI	Mass Spectrometry—Electron Spray Ionization
