# Supplementary material for: Selective Formation, Reactivity, Redox and Magnetic Properties of MnIII and FeIII Dinuclear Complexes with Shortened Salen-Type Schiff Base Ligands
Source: Int J Mol Sci. 2020 Oct 23;21(21):7882. doi: 10.3390/ijms21217882 (PMC7660604; doi:10.3390/ijms21217882)
Supplement: Supplementary file 1 [file ijms-21-07882-s001.pdf]

Supplementary Materials (SM) for:

Selective formation, reactivity, redox and magnetic properties of Mn^{III} and Fe^{III} dinuclear complexes with shortened salen-type Schiff base ligands

Luca Rigamonti, Paolo Zardi, Stefano Carlino, Francesco Demartin, Carlo Castellano, Laura Pigani, Alessandro Ponti, Anna M. Ferretti and Alessandro Pasini

Index

p. S2 **Experimental section (cont.)**

p. S5 **Table S1.** Crystallographic data for H_2sal(*p*-*t*Bu)ben, H_2sal(*p*-CF_3)ben, **2a**·2AcOEt, **2a**·2CH_3CN, **2b**·1.5H_2O and **3c**·2DMF.

p. S7 **Figure S1.** Intermolecular hydrogen bonds in H_2sal(*p*-CF_3)ben.

p. S7 **Figure S2**. Crystal structure of **2a**·2CH_3CN with main atom numbering, and its crystal packing.

p. S8 **Figure S3**. Crystal packing of **2a**·2AcOEt.

p. S8 **Figure S4**. Crystal packing of **3c**·2DMF.

p. S9 **Figure S5**. Crystal packing of **2b**·1.5H_2O.

p. S10 **Figure S6**. CV scans of **3b** recorded in DMF 0.1 M $TBAPF_6$ at 50 mV/s scan rate.

p. S10 **Figure S7**. CV scans of **5a** recorded in DMF 0.1 M $TBAPF_6$ at 50 mV/s scan rate.

p. S11 **Figure S8**. CV scans of **4b** and **6a** recorded in DMF 0.1 M $TBAPF_6$ at 50 mV/s scan rate.

p. S12 **Figure S9**. Temperature dependence of the molar susceptibility of manganese(III) (**3b**, **5a**, **5e, 5f**) and iron(III) (**4b**, **6a**, **6e**) dinuclear compounds.

Experimental section (cont.)

Synthesis of [Mn_2(μ-salmen)$_2$(μ-OMe)$_2$] (3b)

This dark green compound was prepared as **3a** starting from Mn(AcO)$_3$·2H_2O (211.2 mg, 0.79 mmol), H_2salmen (200.1 mg, 0.79 mmol), Et_3N (3 mL) and MeOH (15 mL). Yield: 219.7 mg (80%). Anal (%) calcd for $C_{32}H_{30}Mn_2N_4O_6$·2H_2O (694.54): C 55.34, H 4.35, N 8.10. Found: C 55.40, H 4.72, N 7.96. ESI-MS (MeOH): *m/z* 645 ([M – OMe]$^+$, 100%). IR (KBr): ν_{max}/cm^{-1} 1631 (C=N).

Synthesis of [Mn_2(μ-salmen)$_2$(μ-OH)$_2$] (3c)

This dark green compound was prepared as **3a** starting from Mn(AcO)$_3$·2H_2O (49.1 mg, 0.20 mmol), H_2salmen (50.2 mg, 0.20 mmol), Et_3N (3 mL) and *i*PrOH (15 mL). Yield: 73.9 mg (60%). Anal (%) calcd for $C_{30}H_{26}Mn_2N_4O_6$·5H_2O (738.50): C 48.79, H 4.91, N 7.59. Found: C 48.94, H 4.64, N 7.52. IR (KBr): ν_{max}/cm^{-1} 1625 (C=N).

Synthesis of [Fe_2(μ-salmen)$_2$(μ-OMe)$_2$] (4b)

This dark brown compound was prepared as **4a** starting from $FeCl_3$ (128.2 mg, 0.79 mmol), H_2salmen (201.0 mg, 0.79 mmol), Et_3N (3 mL) and MeOH (15 mL). Yield: 209.2 mg (78%). Anal (%) calcd for $C_{32}H_{30}Fe_2N_4O_6$ (678.34): C 56.66, H 4.46, N 8.26. Found: C 56.33, H 4.73, N 8.13. ESI-MS (MeOH): *m/z* 647 ([M – OMe]$^+$, 60%), 701 ([M + Na]$^+$, 100). IR (KBr): ν_{max}/cm^{-1} 1616 (C=N).

Synthesis of [Fe_2(μ-salmen)$_2$(μ-OH)$_2$] (4c)

This dark green compound was prepared as **4a** starting from $FeCl_3$ (32.0 mg, 0.20 mmol), H_2salmen (50.2 mg, 0.20 mmol), Et_3N (3 mL) and *i*PrOH (15 mL). Yield: 46.7 mg (70%). Anal (%) calcd for $C_{30}H_{26}Fe_2N_4O_6$·H_2O (668.30): C 53.92, H 4.22, N 8.38. Found: C 53.55, H 4.06, N 8.14. ESI-MS (MeOH): *m/z* 633 ([M – OH]$^+$, 10%), 647 (M – 2OH + OMe]$^+$, 100) (OH/OMe exchange in MeOH solution). IR (KBr): ν_{max}/cm^{-1} 1613 (C=N).

Synthesis of [Mn_2(μ-sal(*p*-Me)ben)$_2$(μ-OMe)$_2$] (5b)

This compound was prepared as **5a** starting from Mn(AcO)$_3$·2H_2O (76.5 mg, 0.44 mmol) and H_2sal(*p*-Me)ben (152.0 mg, 0.44 mmol). Yield: 84.8 mg (45%). Anal (%) calcd for $C_{46}H_{42}Mn_2N_4O_6$ (856.74): C 64.50, H 4.94, N 6.54. Found: C 64.87, H 4.80, N 6.54. ESI-MS (MeOH): *m/z* 825 ([M – OMe]$^+$, 100%), 879 ([M + Na]$^+$, 50). IR (KBr): ν_{max}/cm^{-1} 1621 (C=N).

Synthesis of [Mn_2(μ-salben)$_2$(μ-OMe)$_2$] (5c)

This compound was prepared as **5a** starting from Mn(AcO)$_3$·2H_2O (117.3 mg, 0.68 mmol) and H_2salben (220.2 mg, 0.67 mmol). Yield: 104.3 mg (38%). Anal (%) calcd for $C_{44}H_{38}Mn_2N_4O_6$ (828.69): C 63.77, H 4.62, N 6.76. Found: C 63.76, H 4.86, N 6.77. ESI-MS (MeOH): *m/z* 797 ([M – OMe]$^+$, 100%), 851 ([M + Na]$^+$, 40). IR (KBr): ν_{max}/cm^{-1} 1622 (C=N).

Synthesis of [Mn_2(μ-sal(*p*-F)ben)$_2$(μ-OMe)$_2$] (5d)

This compound was prepared as **5a** starting from Mn(AcO)$_3$·2H_2O (77.5 mg, 0.45 mmol) and H_2sal(*p*-F)ben (153.1 mg, 0.44 mmol). Yield: 62.8 mg (33%). Anal (%) calcd for $C_{44}H_{36}F_2Mn_2N_4O_6$ (864.67): C 61.12, H 4.20, N 6.48. Found: C 60.75, H 4.30, N 6.41. ESI-MS (MeOH): not soluble. IR (KBr): ν_{max}/cm^{-1} 1622 (C=N).

Synthesis of [Mn_2(μ-sal(*p*-Cl)ben)$_2$(μ-OMe)$_2$] (5e)

This compound was prepared as **5a** starting from Mn(AcO)$_3$·2H_2O (169.5 mg, 0.97 mmol) and H_2sal(*p*-Cl)ben (355.5 mg, 0.99 mmol). Yield: 189.8 mg (43%). Anal (%) calcd for $C_{44}H_{36}Cl_2Mn_2N_4O_6$·$H_2O$ (915.59): C 57.72, H 4.18, N 6.12. Found: C 57.55, H 4.23, N 6.04. ESI-MS (MeOH): *m*/*z* 865 ([M – OMe]$^+$, 100%), 897 (([M + H]$^+$, 95), 919 ([M + Na]$^+$, 10). IR (KBr): ν_{max}/cm^{-1} 1620 (C=N).

Synthesis of [Mn_2(μ-sal(*p*-CF_3)ben)$_2$(μ-OMe)$_2$] (5f)

This compound was prepared as **5a** starting from Mn(AcO)$_3$·2H_2O (69.1 mg, 0.40 mmol) and H_2sal(*p*-CF_3)ben (152.6 mg, 0.38 mmol). Yield: 86.5 mg (47%). Anal (%) calcd for $C_{46}H_{36}F_6Mn_2N_4O_6$ (964.68): C 57.27, H 3.76, N 5.81. Found: C 57.34, H 3.64, N 5.73. ESI-MS (MeOH): not soluble. IR (KBr): ν_{max}/cm^{-1} 1621 (C=N).

Synthesis of [Mn_2(μ-sal(*p*-NO_2)ben)$_2$(μ-OMe)$_2$] (5g)

This compound was prepared as **5a** starting from Mn(AcO)$_3$·2H_2O (34.0 mg, 0.13 mmol) and H_2sal(*p*-NO_2)ben (50.0 mg, 0.13 mmol). Yield: 57.3 mg (53%). Anal (%) calcd for $C_{44}H_{36}Mn_2N_6O_{10}$·MeOH (964.68): C 56.85, H 4.24, N 8.84. Found: C 56.41, H 3.92, N 8.82. ESI-MS (MeOH): not soluble. IR (KBr): ν_{max}/cm^{-1} 1619 (C=N).

Synthesis of [Fe_2(μ-sal(*p*-Me)ben)$_2$(μ-OMe)$_2$] (6b)

The synthesis of this compound was performed as **6a** different times starting from Fe(NO_3)$_3$·9H_2O or $FeCl_3$ and H_2sal(*p*-Me)ben in equimolar ratio, but little solid was left after mixing all times, and precipitation of the reaction solution with H_2O or *i*Pr_2O always yielded a red solid, whose IR spectrum invariably presented the N–H stretching of the hydrolysed sal(*p*-Me)ben^{2-} ligand to salim$^-$ at 3305 cm^{-1}, together with the C=O stretching of the free *para*-methylbenzaldehyde at 1700 cm^{-1}. The C=N stretching at 1617 cm^{-1} is also present.

Synthesis of [Fe_2(μ-salben)$_2$(μ-OMe)$_2$] (6c)

This compound was prepared as **6a** starting from Fe(NO_3)$_3$·9H_2O (124.4 mg, 0.308 mmol) and H_2salben (101.9 mg, 0.308 mmol). Yield: 73.3 mg (54%). Anal (%) calcd for $C_{44}H_{38}Fe_2N_4O_6$·MeOH·H_2O (880.557): C 61.38, H 5.04, N 6.36. Found: C 61.32, H 4.66, N 6.36. IR (KBr): ν_{max}/cm^{-1} 1614 (C=N). ESI-MS (MeOH): *m*/*z* 799 ([M – OMe]$^+$, 100%), 853 ([M + Na]$^+$, 30), 881 (M + MeOH + H_2O + H]$^+$. 40). ***Warning!*** The reaction mixture was left under stirring only 1 h at room temperature and then the title compound was isolated as dark red solid by filtration. For longer times, as for **6b**, the solid slowly disappeared leaving a red solution; the addition of water led to the precipitation of a brown-red solid, which revealed the presence of one hydrolysed salben^{2-} ligand to two salim$^-$, as evidenced in the infrared spectrum by the N–H stretching at 3302 cm^{-1} and the C=O stretching of the free benzaldehyde at 1697. The C=N stretching at 1616 cm^{-1} is also present. ESI-MS (MeOH): *m*/*z* 606 ([Fe_2(salmp)(salim)(OMe)]$^+$, 100%), 749 ([Fe_2(salmp)(salim)$_2$(OMe) + Na]$^+$, 50).

Synthesis of [Fe_2(μ-sal(*p*-F)ben)$_2$(μ-OMe)$_2$] (6d)

The synthesis of this compound was performed as **6a** different times starting from Fe(NO_3)$_3$·9H_2O or $FeCl_3$ and H_2sal(*p*-F)ben, but in all cases little solid was left after mixing for 1 h, and

precipitation of the reaction solution with H_2O or *i*Pr_2O always yielded a red solid whose IR spectrum invariably presented the N–H stretching of the hydrolysed sal(*p*-F)ben^{2-} ligand to $salim^{-}$ at 3313 cm^{-1}, together with the C=O stretching of the free *p*-F-benzaldehyde at 1690 cm^{-1}. The C=N stretching at 1615 cm^{-1} is also present. ESI-MS (MeOH): *m*/*z* 606 ($[Fe_2(salmp)(salim)(OMe)]^+$, 100%), 749 ($[Fe_2(salmp)(salim)_2(OMe) + Na]^+$, 10), 883 ($[Fe_2(salmp)(salim)_2(OMe)$ + *p*-F-benzaldehyde·MeOH + $H]^+$, 60), where *p*-F-benzaldehyde·MeOH = hemiacetal.

Synthesis of [Fe_2(μ-sal(*p*-Cl)ben)$_2$(μ-OMe)$_2$] (6e)

This compound was prepared as **6a** starting from $Fe(NO_3)_3·9H_2O$ (357.4 mg, 0.885 mmol) and H_2sal(*p*-Cl)ben (315.3 mg, 0.864 mmol). Yield: 156.1 mg (34%). Anal (%) calcd for $C_{44}H_{36}Cl_2Fe_2N_4O_6·2MeOH·H_2O$ (981.49): C 56.29, H 4.72, N 5.71. Found: C 56.68, H 4.37, N 5.72. IR (KBr): ν_{max}/cm^{-1} 1614 (C=N). ESI-MS (MeOH) on freshly-prepared solution: *m*/*z* 867 ($[M - OMe]^+$, 100%), 899 ($[M + 1]^+$, 20), 921 ($[M + Na]^+$, 45). ESI-MS (MeOH) on aged solution for 1 day: *m*/*z* 656 ([Fe_2(sal(*p*-Cl)ben)(salim)$(OMe)_2]^+$, 25%), 799 ([Fe_2(sal(*p*-Cl)ben)$(salim)_2(OMe)_2$ + $Na]^+$, 100), 867 ($[M - OMe]^+$, 10), 899 ($[M + 1]^+$, 20), 921 ($[M + Na]^+$, 10).

Synthesis of [Fe_2(μ-sal(*p*-CF_3)ben)$_2$(μ-OMe)$_2$] (6f)

The synthesis of this compound was performed as **6a** different times starting from $Fe(NO_3)_3·9H_2O$ or $FeCl_3$ and H_2sal(*p*-CF_3)ben, but in all cases little solid was left after mixing for 1 h, and precipitation of the reaction solution with H_2O or *i*Pr_2O always yielded a red solid, whose IR spectrum invariably presented the N–H stretching of the hydrolysed sal(*p*-CF_3)ben^{2-} ligand to $salim^{-}$ at 3296 cm^{-1}, together with the C=O stretching of the free *p*-CF_3-salicylaldehyde at about 1700^{-1}. The C=N stretching at 1616 cm^{-1} is also present.

Synthesis of [Fe_2(μ-sal(*p*-NO_2)ben)$_2$(μ-OMe)$_2$] (6g)

The synthesis of this compound was performed as **6a** different times starting from $Fe(NO_3)_3·9H_2O$ or $FeCl_3$ and H_2sal(*p*-NO_2)ben, but in all cases little solid was left after mixing, and precipitation of the reaction mixture with H_2O or *i*Pr_2O always yielded a solid, whose IR spectrum invariably presented the N–H stretching of the hydrolysed sal(*p*-NO_2)ben^{2-} ligand to $salim^{-}$ at 3316 cm^{-1}, together with the C=O stretching of the free *p*-NO_2-salicylaldehyde at 1724 cm^{-1}. The C=N stretching at 1616 cm^{-1} is also present.

Table S1. Crystallographic data for H_2sal(*p*-*t*Bu)ben, H_2sal(*p*-CF_3)ben, **2a**·2AcOEt, **2a**·2CH_3CN, **2b**·1.5H_2O and **3c**·2DMF.

	H_2sal(*p*-*t*Bu)ben	H_2sal(*p*-CF_3)ben	**2a**·2AcOEt
Crystal Data			
Moiety formula	$C_{25}H_{17}N_2O_2$	$C_{22}H_{17}F_3N_2O_2$	$C_{42}H_{30}Fe_2N_4O_6$ ·$2C_4H_8O_2$
M	377.40	398.37	974.61
Crystal system	orthorhombic	orthorhombic	triclinic
Space group	*Pnma* (n. 62)	*Pna2₁* (n. 33)	*P–1* (n. 2)
a / Å	11.0752(16)	9.806(2)	9.914(2)
b / Å	19.832(3)	14.949(3)	10.524(2)
c / Å	9.9100(15)	13.214(3)	10.892(2)
α / °	90	90	92.53(3)
β / °	90	90	100.72(3)
γ / °	90	90	90.38(3)
V / $Å^3$	2176.7(6)	1937.0(7)	1115.4(4)
Z	4	4	1
ρ_{calc} / g cm^{-3}	1.152	1.366	1.451
μ / mm^{-1}	0.063	0.108	0.716
Colour, habit	colourless, prism	colourless, prism	brown, pseudoprism
Dimensions / mm	0.25 × 0.15 × 0.08	0.25 × 0.10 × 0.03	0.12 × 0.08 × 0.05
Data Collection			
Temperature / K	292(2)	294(2)	294(2)
radiation λ / Å	Mo Kα, 0.71073	Mo Kα, 0.71073	Mo Kα, 0.71073
$2\theta_{max}$ / º	52.9	36.5	57.9
Measured reflections	14942	5048	8669
Independent reflections	2314	1386	5143
Reflections [I>2σ(I)]	1473	1303	3583
R_{int}	0.038	0.021	0.030
Data refinement			
R_1, wR_2 [I>2σ(I)]	0.0605, 0.1890	0.0315, 0.0821	0.0474, 0.1144
R_1, wR_2 [all data]	0.0860, 0,2144	0.0344, 0.0848	0.0867, 0.1381
Goodness of fit *S*	0.965	1.045	1.045
Flack parameter	–	0.2(4)	–
Parameters, restraints	158, 0	270, 1	298, 0
$\Delta\rho_{max}$, $\Delta\rho_{min}$ / e $Å^{-3}$	0.27, –0.17	0.17, –0.11	0.60, –0.57

Table S1 (cont.)

	**2a**·2CH3CN	**2b**·1.5H2O	**3c**·2DMF
Crystal Data			
Moiety formula	$C_{42}H_{30}Fe_2N_4O_6$ ·$2C_2H_3N$	$C_{36}H_{30}Fe_2N_4O_6$ ·$1.5H_2O$	$C_{30}H_{26}Mn_2N_4O_6$ ·$2C_3H_7NO$
M	880.51	753.36	794.62
Crystal system	triclinic	orthorhombic	triclinic
Space group	*P–1* (n. 2)	$Pmc2_1$ (n. 26)	*P–1* (n. 2)
a / Å	9.807(2)	17.4994(18)	9.7228(14)
b / Å	10.787(2)	10.5522(11)	9.8409(14)
c / Å	11.396(2)	18.5062(19)	10.8652(16)
α / °	70.17(3)	90	65.480(10)
β / °	65.23(3)	90	67.880(10)
γ / °	88.98(3)	90	83.170(10)
V / $Å^3$	1018.8(5)	3417.3(6)	875.4(2)
Z	1	4	1
ρ_{calc} / g cm^{-3}	1.435	1.464	1.507
μ / mm^{-1}	0.770	0.906	0.783
Colour, habit	brown, prism	brown, prism	brown, pseudoprism
Dimensions / mm	0.15 × 0.10 × 0.08	0.15 × 0.06 × 0.05	0.15 × 0.07 × 0.05
Data Collection			
Temperature / K	293(2)	294(2)	294(2)
radiation λ / Å	Mo Kα, 0.71073	Mo Kα, 0.71073	Mo Kα, 0.71073
$2\theta_{max}$ / º	46.2	57.4	58.3
Measured reflections	5328	27448	8175
Independent reflections	2851	8855	4337
Reflections [I>2σ(I)]	2415	5677	3262
R_{int}	0.020	0.049	0.096
Data refinement			
R_1, wR_2 [I>2σ(I)]	0.0294, 0.0678	0.0400, 0.0898	0.0333, 0.0860
R_1, wR_2 [all data]	0.0364, 0.0705	0.0771, 0.1031	0.0587, 0.1033
Goodness of fit *S*	0.981	0.947	1.087
Flack parameter	–	–0.009(8)	–
Parameters, restraints	271, 0	487, 1	247, 0
$\Delta\rho_{max}$, $\Delta\rho_{min}$ / e $Å^{-3}$	0.18, –0.22	0.42, –0.29	0.41, –0.64

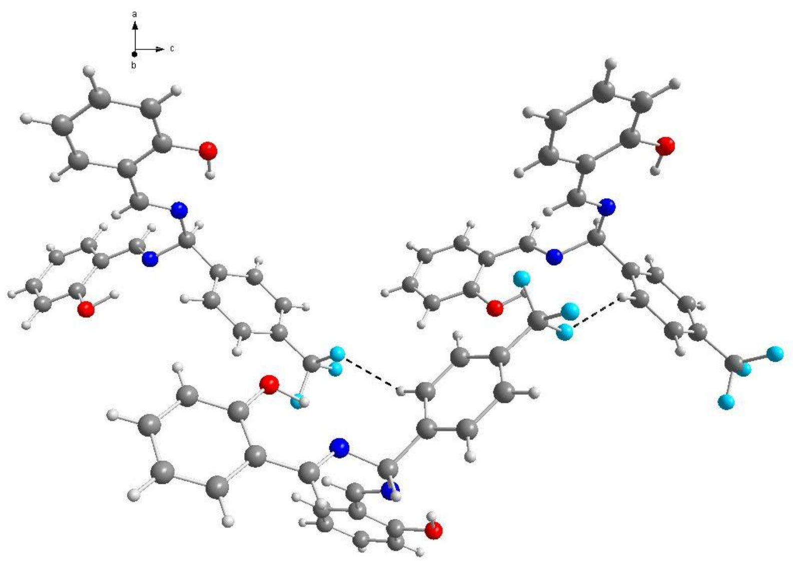

Figure S1. Intermolecular hydrogen bonds in H_2sal(*p*-CF_3)ben. Colour code: O = red, N = blue, C = grey, H = white, F = turquoise.

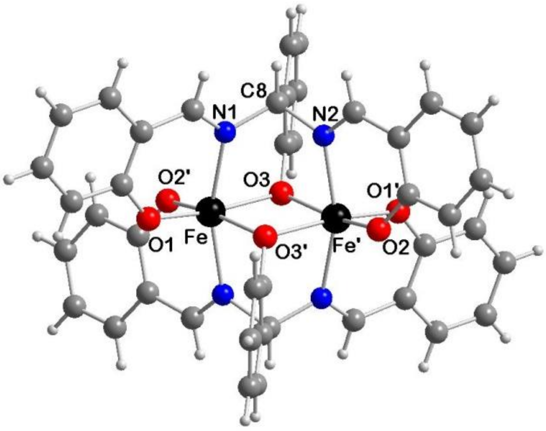

(a)

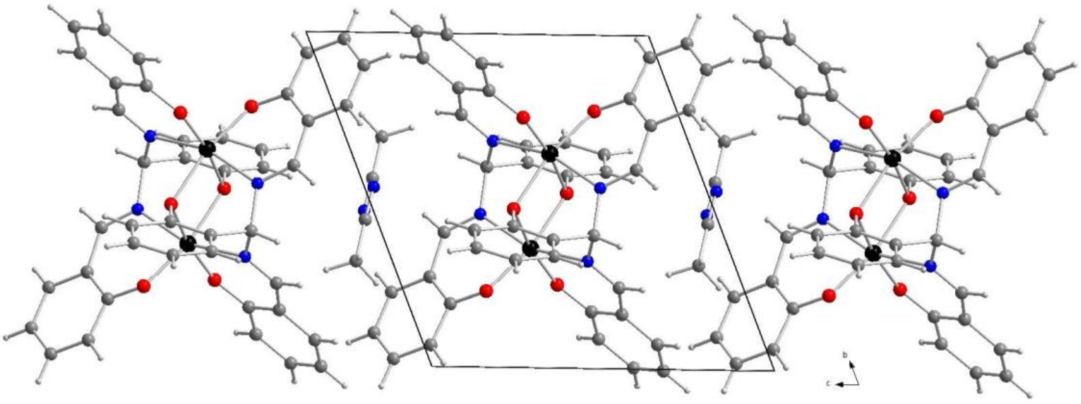

(b)

Figure S2. (a) Crystal structure of **2a**·2CH_3CN with main atom numbering and (b) its crystal packing; colour code: Fe = black, O = red, N = blue, C = grey, H = white.

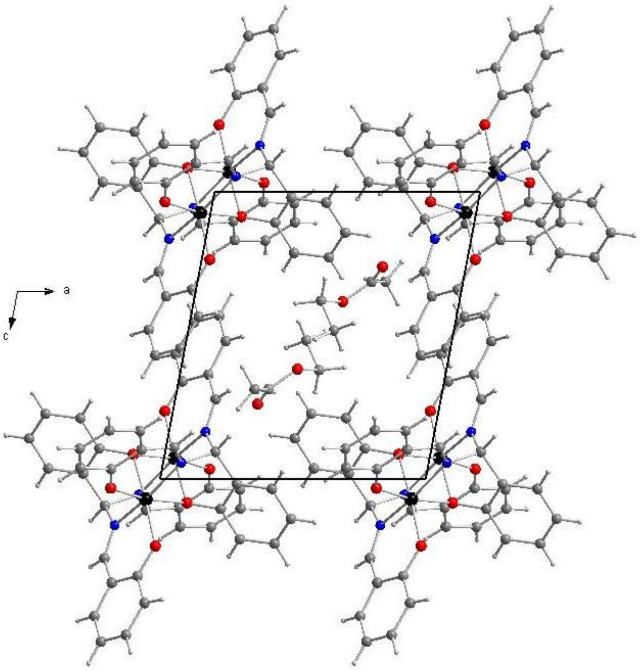

Figure S3. Crystal packing of **2a**·2AcOEt; colour code: Fe = black, O = red, N = blue, C = grey, H = white.

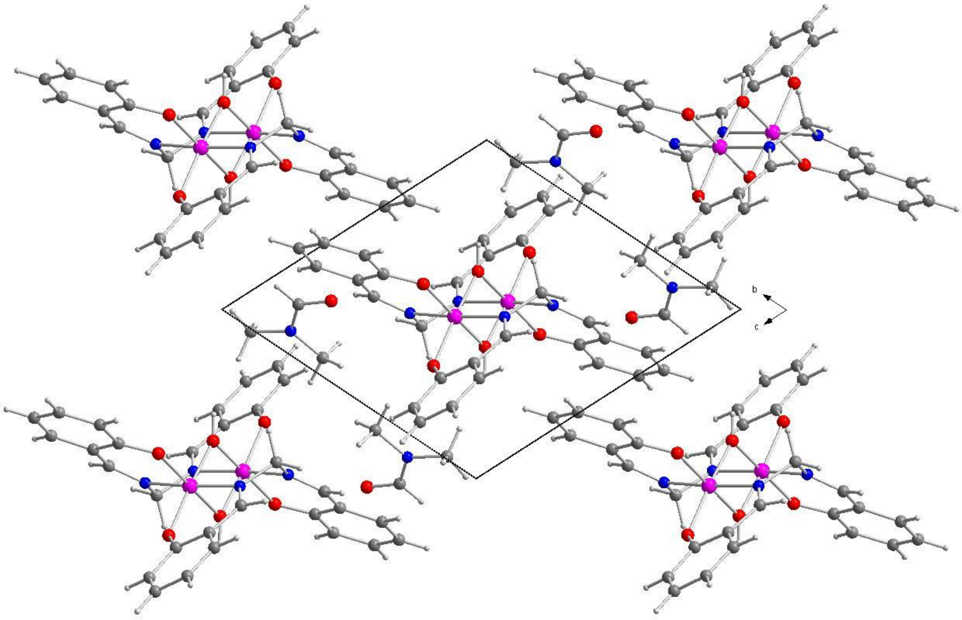

Figure S4. Crystal packing of **3c**·2DMF; colour code: Mn = violet, O = red, N = blue, C = grey, H = white.

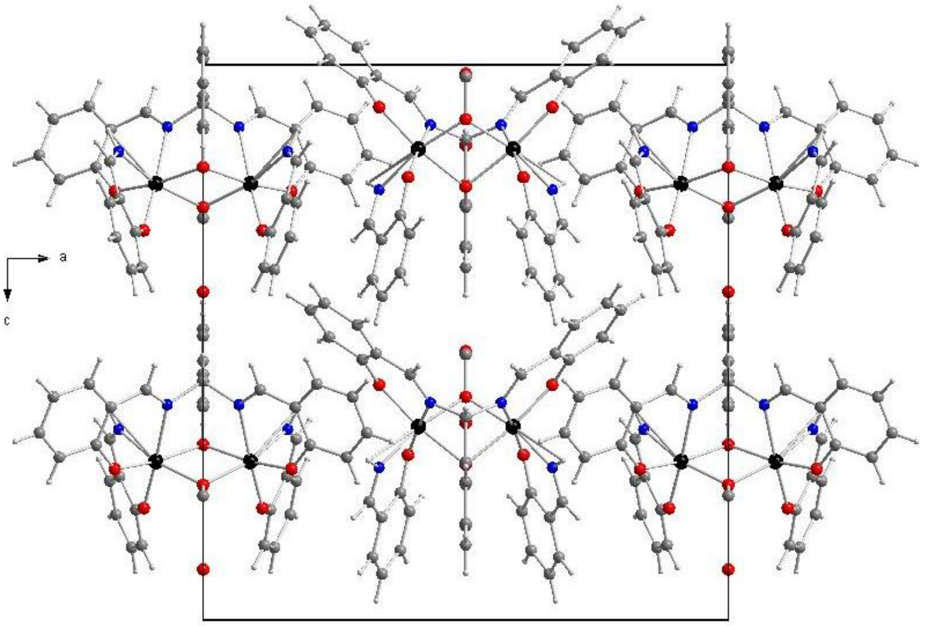

Figure S5. Crystal packing of **2b**·1.5H_2O; colour code: Fe = black, O = red, N = blue, C = grey, H = white.

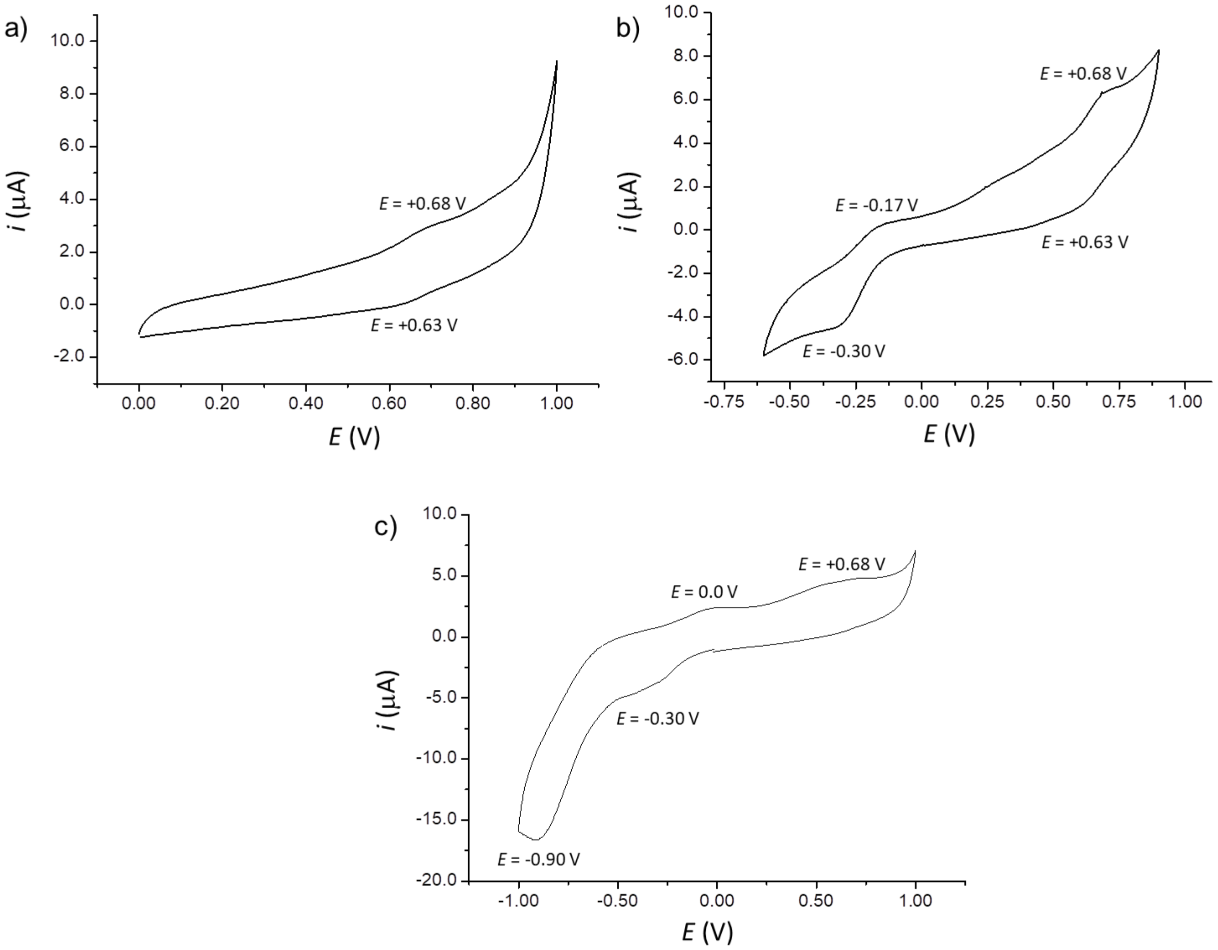

Figure S6. CV scans of **3b** recorded in DMF 0.1 M $TBAPF_6$ at 50 mV/s scan rate; potentials measured *vs* Ag/AgCl, 3 M KCl reference electrode.

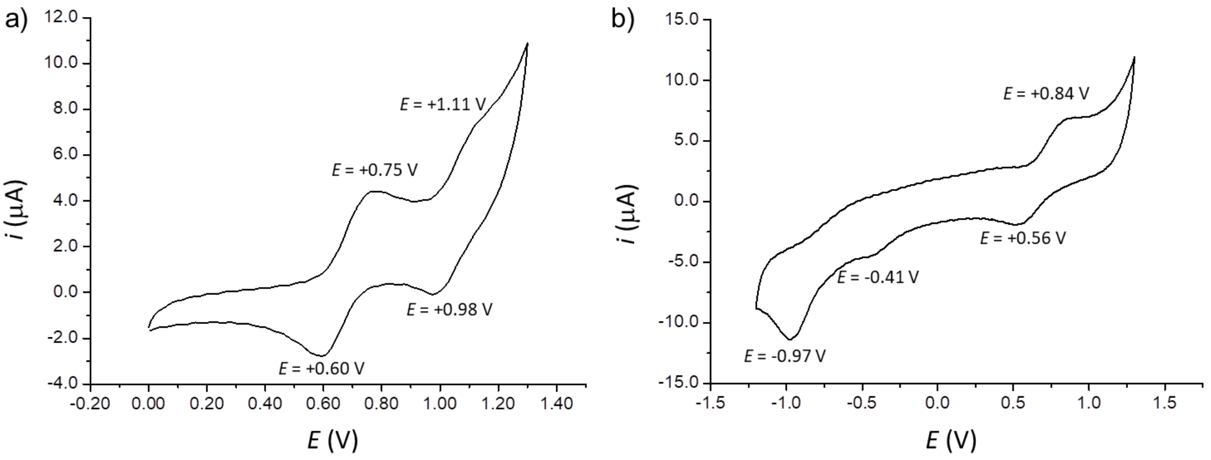

Figure S7. CV scans of **5a** recorded in DMF 0.1 M $TBAPF_6$ at 50 mV/s scan rate; potentials measured *vs* Ag/AgCl, 3 M KCl reference electrode.

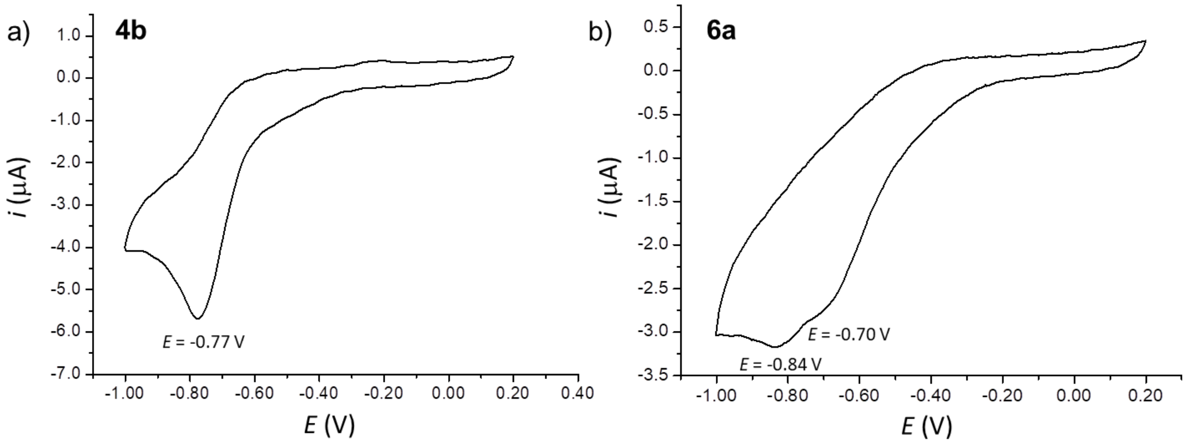

Figure S8. CV scans of a) **4b** and b) **6a** recorded in DMF 0.1 M $TBAPF_6$ at 50 mV/s scan rate; potentials measured *vs* Ag/AgCl, 3 M KCl reference electrode.

4b

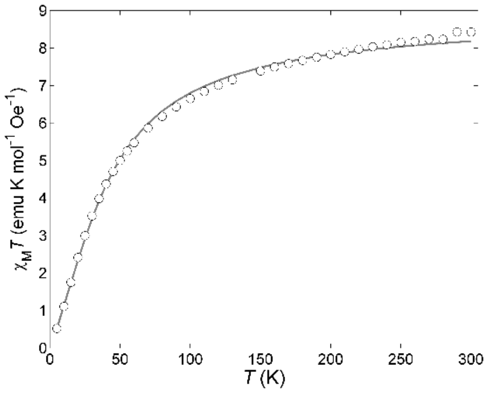

3b

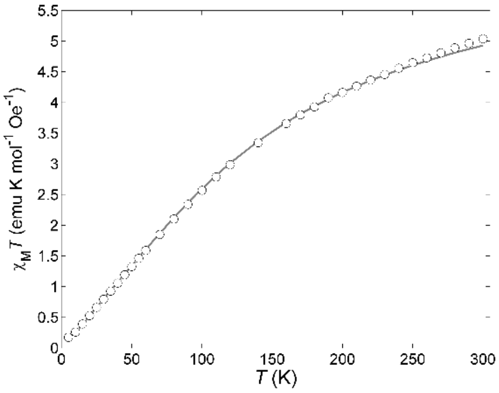

6a

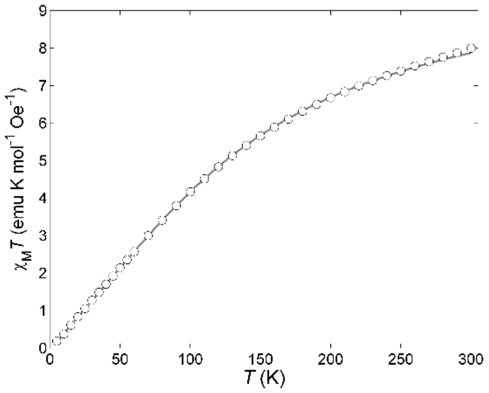

5a

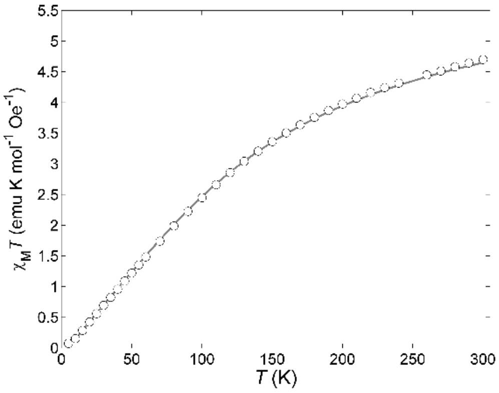

6e

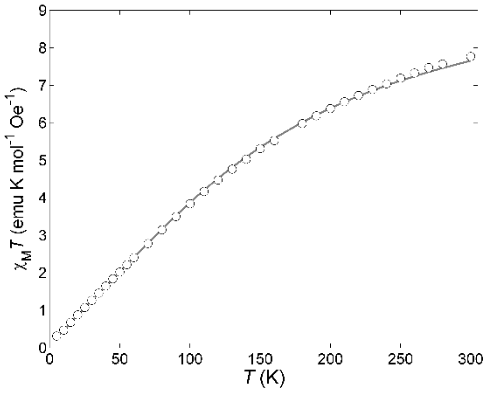

5e

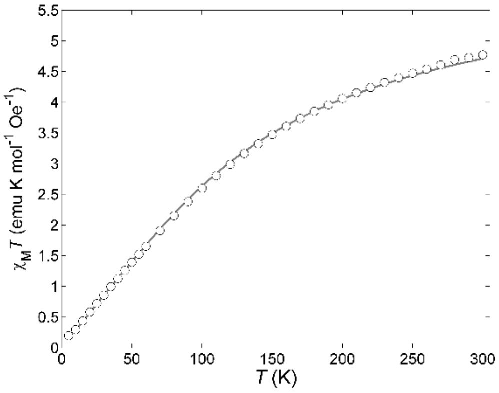

5f

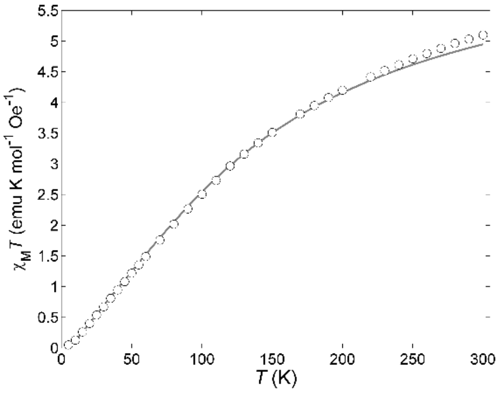

Figure S7. Temperature dependence of the molar susceptibility of iron(III) (**4b**, **6a**, **6e**) and manganese(III) (**3b**, **5a**, **5e, 5f**) dinuclear compounds.
